# New group of transmembrane proteins associated with desiccation tolerance in the anhydrobiotic midge *Polypedilum vanderplanki*

**DOI:** 10.1038/s41598-020-68330-6

**Published:** 2020-07-15

**Authors:** Taisiya A. Voronina, Alexander A. Nesmelov, Sabina A. Kondratyeva, Ruslan M. Deviatiiarov, Yugo Miyata, Shoko Tokumoto, Richard Cornette, Oleg A. Gusev, Takahiro Kikawada, Elena I. Shagimardanova

**Affiliations:** 1 Extreme Biology laboratory, Institute of Fundamental Medicine and Biology, Kazan Federal University, Kazan, Russia; 2 Division of Biotechnology, Institute of Agrobiological Sciences, National Institute of Agriculture and Food Research Organization (NARO), Tsukuba, Japan; 3 Graduate School of Frontier Sciences, The University of Tokyo, Kashiwa, Japan; 4 KFU-RIKEN Translational Genomics Unit, RIKEN Cluster for Science, Technology and Innovation Hub, RIKEN, Yokohama, Japan; 5 Laboratory for Transcriptome Technology, RIKEN Center for Integrative Medical Sciences, RIKEN, Yokohama, Japan

**Keywords:** Membrane proteins, Gene expression, Molecular biology

## Abstract

Larvae of the sleeping chironomid *Polypedilum vanderplanki* are known for their extraordinary ability to survive complete desiccation in an ametabolic state called “anhydrobiosis”. The unique feature of *P. vanderplanki* genome is the presence of expanded gene clusters associated with anhydrobiosis. While several such clusters represent orthologues of known genes, there is a distinct set of genes unique for *P. vanderplanki*. These include Lea-Island-Located (LIL) genes with no known orthologues except two of LEA genes of *P. vanderplanki*, *PvLea1* and *PvLea3*. However, PvLIL proteins lack typical features of LEA such as the state of intrinsic disorder, hydrophilicity and characteristic LEA_4 motif. They possess four to five transmembrane domains each and we confirmed membrane targeting for three PvLILs. Conserved amino acids in PvLIL are located in transmembrane domains or nearby. PvLEA1 and PvLEA3 proteins are chimeras combining LEA-like parts and transmembrane domains, shared with PvLIL proteins. We have found that *PvLil* genes are highly upregulated during anhydrobiosis induction both in larvae of *P. vanderplanki* and *P. vanderplanki-*derived cultured cell line, Pv11. Thus, *PvLil* are a new intriguing group of genes that are likely to be associated with anhydrobiosis due to their common origin with some LEA genes and their induction during anhydrobiosis.

## Introduction

Anhydrobiosis is the ability of an organism to survive complete desiccation in the ametabolic state. Animals able to enter anhydrobiosis at least at some life stages are found in four invertebrate phyla, namely tardigrades, rotifers, nematodes and arthropods^1,2^. Damaging effects of desiccation in these animals are mitigated via interplay of numerous protective mechanisms, including the formation of biological glass (vitrification), “molecular shield” and anti-aggregation activity of some proteins and enhanced antioxidant activity^3–5^. Intrinsically disordered proteins (IDP’s) frequently participate at least in some of the protective mechanisms related to desiccation tolerance^5–9^. Anhydrobiotic animals share also such features as a small size which is typically less than 5 mm and an absence of internal skeletons, at least on anhydrobiotic life stages. This may be related to physical stresses associated with body shrinking during water loss^10^. Larvae of the sleeping chironomid *Polypedilum vanderplanki* (Diptera) reaching 7 mm in length are the largest and the most complex organisms able to enter anhydrobiosis^11^. This midge inhabits semi-arid rocks in Nigeria, and its larvae represent the only stage of the life cycle that is capable of enduring the desiccation at the onset of the dry season^12^.

The induction of the anhydrobiotic state in *P. vanderplanki* is a gradual process mediated by the replacement of intracellular water with non-reducing sugar trehalose and the accumulation of bioprotectants, such as heat-shock proteins, antioxidant enzymes and the late embryogenesis abundant (LEA) proteins^13^. LEA proteins are believed to act as molecular shields and LEA proteins of *P. vanderplanki* (PvLEA) were shown to prevent the aggregation of protein molecules during water loss and contribute to vitrification^14–16^.

*PvLea* genes and many other key anhydrobiosis-induced genes are localized in genome of *P. vanderplanki* in several compact genomic clusters, named ARId (anhydrobiosis-related island of genes)^17^. In many cases, the genes with a key involvement in the acquisition of desiccation tolerance are represented in a genome by multiple copies, and it seems that duplication of essential genes in the evolutionary process is a single-species attribute of *P. vanderplanki*^17^. In the *P. vanderplanki* genome, 27 *Lea* genes (*PvLea*) were identified, and the majority of them are localized in a single genomic cluster. In this cluster we also found 13 tandemly located paralogous genes that have no known orthologues in other species. Taking into account their co-localization with *PvLea* genes, the newly observed genes were named “LEA-island located” (*Lil*) genes.

Here we made the first detailed analysis of *PvLil* genes and their relation to PvLEA. We implemented an in silico approach to reveal whether PvLIL proteins possess typical LEA characteristics such as the state of intrinsic disorder, hydrophilicity and the typical LEA_4 motif. We identified conserved amino acid residues and domains typical of PvLILs to get first glimpses into their possible functions, confirming the targeting of some PvLIL proteins in vivo. We also determined expression profiles of *PvLil* genes in larvae of *P. vanderplanki* and the *P. vanderplanki*-derived Pv11 cell line to reveal their possible association with the induction of anhydrobiosis. Since the larval stage is the only stage of life cycle of *P. vanderplanki* that is able to enter anhydrobiosis, we compared *PvLil* expression between different life cycle stages.

## Results

### *PvLil* are localized in the same ARId cluster as *PvLea* genes are clustered within the *PvLea* on a phylogenetic tree

**Figure 1:**
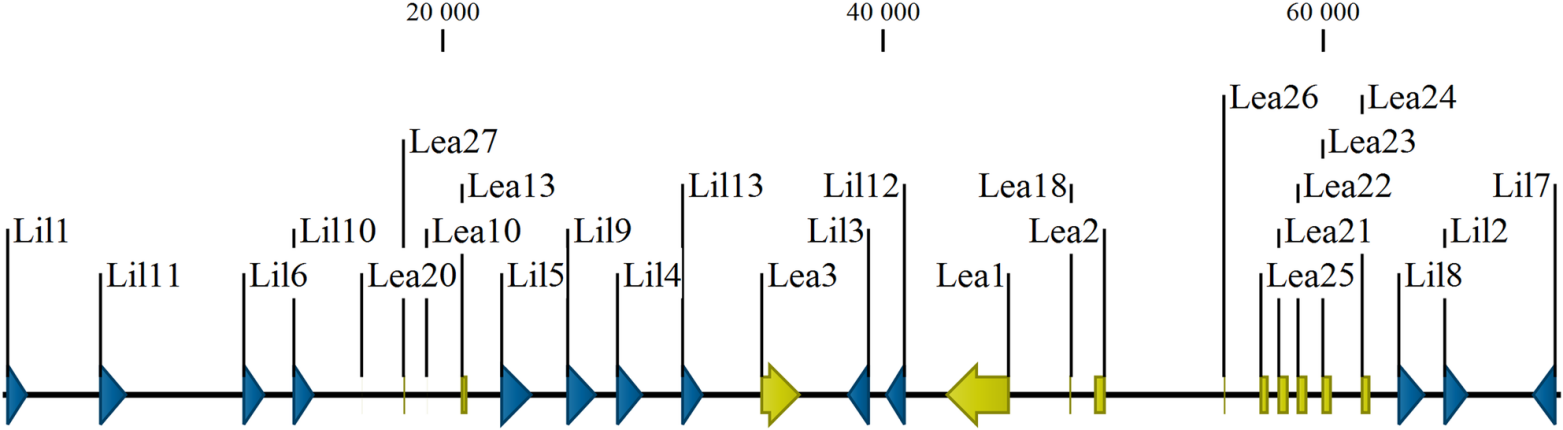
The localization of *PvLea* and *PvLil* genes in the ARId1. Schematic of a part of the ARId1 region (nucleotides 27,300:98,100) incorporating all *PvLil* genes. Genes are represented by arrows and boxes, whose colour indicates different types of genes: *PvLil* (blue) and *PvLea* (yellow). The prefix “Pv” and annotations of six genes that are not related to LEA were removed for clarity.

**Figure 2:**
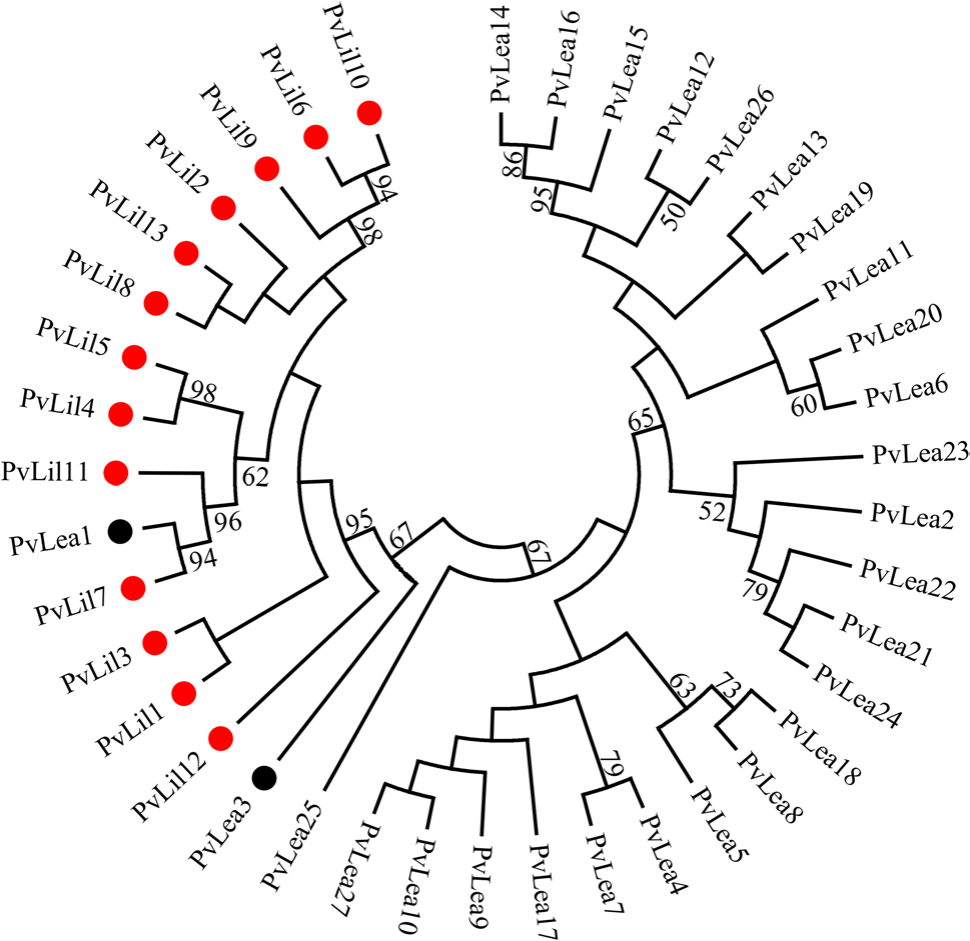
The phylogenetic tree of *PvLil* and *PvLea* genes.

One of the most interesting features of the *P. vanderplanki* genome is the presence of compact gene clusters called ARIds^17^. These clusters are mostly populated by highly duplicated paralogous genes which are absent in the non-anhydrobiotic related species *P. nubifer*. Many genes in ARIds are upregulated during induction of anhydrobiosis in *P. vanderplanki*^17^. For example, ARId1 and ARId2 incorporate 26 out of 27 genes of LEA proteins of *P. vanderplanki* (*PvLea*), and 23 *PvLea* genes are induced during desiccation more than threefold^17^. LEA proteins are widespread in anhydrobiotic organisms from different taxa and one of the best-known effectors of mechanisms ensuring water deficit tolerance. Here we describe for the first time the new group of genes, which are co-located with *PvLea* genes in ARId1. Genes of a new group are located tandemly in between *PvLea* genes (Fig. 1) and referred to as “LEA-Island Located” (LIL). We hypothesized that these genes are related to *PvLea* genes since they fall into one clade with *PvLea1* and *PvLea3* on a maximum likelihood phylogenetic tree (Fig. 2).

The phylogenetic tree was constructed by using the Maximum Likelihood method based on the General Time Reversible model in MEGA7^50^. The consensus tree with the highest log likelihood (−29,560.8924) is shown. The initial tree for the heuristic search was obtained by applying the Neighbor-Joining method to a matrix of pairwise distances estimated using the Maximum Composite Likelihood (MCL) approach. The analysis involved 40 nucleotide sequences. Sequences of *PvLil* genes were verified by cloning and Sanger sequencing, resulting sequences are shown in Supplementary Data 1. There were a total of 2,229 positions in the final dataset. Red dots denote *PvLil* genes. *PvLea1* and *PvLea3* genes are indicated by black dots. Bootstrap values with 1,000 replicates are shown at the nodal branches (cutoff value = 50%).

These genes are specific for *P. vanderplanki* since no hits were found in the NCBI non-redundant gene sequences database in the BLASTN and discontinuous MegaBLAST programs, except for *PvLea1* and *PvLea3* genes from the *P. vanderplanki* genome. This similarity reinforces the connection of *PvLil* genes to *PvLea.* Next, we investigated with the MegaBLAST algorithm whether *P. vanderplanki* genome possesses other previously unannotated members of this group and found none in our published PvScaf 0.9 genome assembly, which is publicly available in the MidgeBase database (https://bertone.nises-f.affrc.go.jp/midgebase/). Thus, our data suggest that this group is restricted to 13 members. HMMER analysis also does not yield any matches of amino acid sequences related to known protein families in PvLIL proteins.

### PvLIL proteins are more hydrophobic and less disordered than PvLEA.

We translated the longest open reading frames of all 13 PvLil genes into protein sequences and determined their biophysical characteristics using the ProtParam service (https://web.expasy.org/protparam/)^18^ (Table 1). The predicted molecular mass of PvLIL proteins varies from 25.9 up to 33.8 kDa. On average, all 13 PvLIL proteins are hydrophobic: values of their grand average hydropathy index (GRAVY) vary from 0.129 to 0.872. It was found that PvLIL proteins are folded: their fold index values are positive and range from 0.27 to 0.50 (Table 1). Isoelectric point values are between 4.87 and 9.14.

**Table 1:** The properties of amino acid sequences of PvLIL proteins.

Protein	GenBank accession number	Length	Mass, kDa	GRAVY index	Fold index	pI
PvLIL1	MT043332	252	29.2	0.34	0.34	8.47
PvLIL2	MT043333	245	28.3	0.32	0.33	8.40
PvLIL3	MT043334	229	25.9	0.87	0.50	5.41
PvLIL4	MT043335	232	27.1	0.62	0.42	5.94
PvLIL5	MT043336	284	32.9	0.13	0.27	8.02
PvLIL6	MT043337	242	27.8	0.51	0.39	6.19
PvLIL7	MT043338	233	26.87	0.78	0.46	5.20
PvLIL8	MT043339	232	26.6	0.44	0.35	9.18
PvLIL9	MT043340	237	27.2	0.38	0.34	8.97
PvLIL10	MT043341	239	27.7	0.53	0.40	7.70
PvLIL11	MT043342	296	33.8	0.2	0.29	5.90
PvLIL12	MT043343	256	29.2	0.62	0.41	4.87
PvLIL13	MT043344	230	26.3	0.41	0.33	9.14

Data on molecular weight, theoretical isoelectric point (pI) and grand average of hydropathy (GRAVY) were obtained via analysis of sequences of proteins corresponding to *PvLil* genes by the ProtParam tool^18^. Fold index values were obtained via the FoldIndex service^52^.

**Figure 3:**
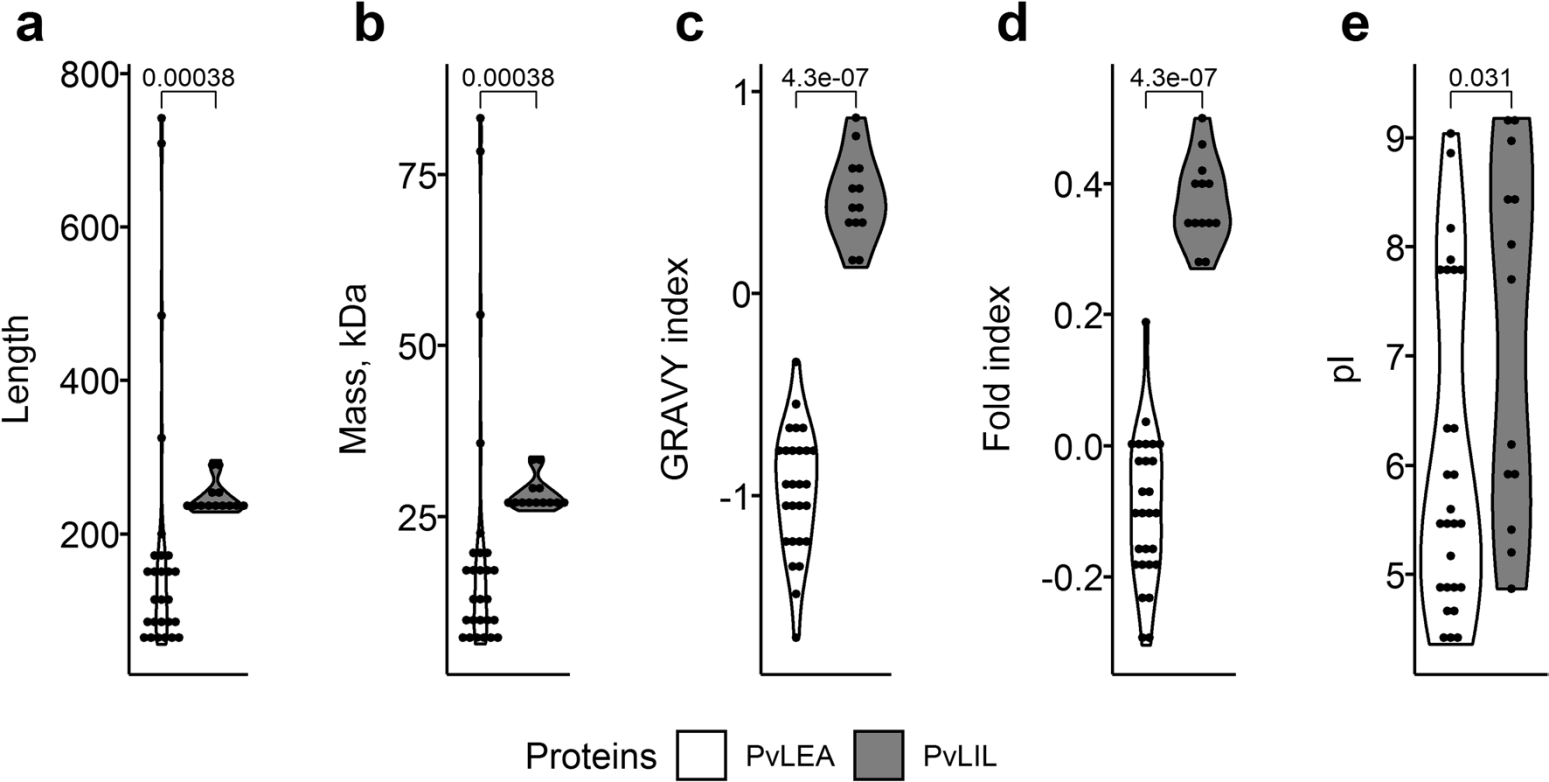
Properties of PvLIL proteins in comparison to PvLEA proteins. (**a**) Length of PvLIL and PvLEA proteins. (**b**) Predicted molecular mass of PvLIL and PvLEA proteins. (**c**) GRAVY index corresponding to the average protein hydrophobicity in PvLIL and PvLEA proteins. Increased GRAVY index values reflect increased hydrophobicity of PvLIL in comparison to PvLEA proteins. (**d**) Diagram of fold index in PvLIL and PvLEA proteins, reflecting whether protein is intrinsically disordered or not (negative and positive values, respectively). In contrast to typical PvLEA proteins, PvLIL proteins are not intrinsically disordered. The positive fold index of some PvLEA reflects the presence of folded domains in these proteins. (**e**) Predicted values of isoelectric point for PvLIL and PvLEA proteins. Molecular mass, GRAVY index and isoelectric point were predicted with ProtParam service^18^, fold index was predicted with FoldIndex^52^. Values of corresponding characteristics are plotted along y-axis. *p* values on the top of each plot were obtained in two-sided Wilcoxon test; n = 13 and 27 for PvLIL and PvLEA, respectively. Continuity correction was introduced to compensate for the presence of tied values; alternative *p* values obtained in 1,000 test replicates with the introduction of random noise were lower than those obtained using correction (see Methods). Data for PvLEA proteins were taken from^19^ and reproduced using the same bioinformatic tools. Black circles depict values of corresponding characteristics for individual proteins.

PvLIL proteins significantly differ from PvLEA in these characteristics as we can conclude from the comparison with previously published PvLEA protein data^19^. PvLIL proteins are significantly longer and have higher predicted molecular mass (Fig. 3; the *p *value in both cases is 0.00038). Here and below in this paragraph p-values were computed in the two-sided Wilcoxon test with continuity correction; n = 13 and 27 for PvLIL and PvLEA, respectively. These characteristics are more variable among PvLEA proteins and the three longest PvLEA (PvLEA1, PvLEA3 and PvLEA5) are much longer than any of the PvLIL proteins (Fig. 3). Most importantly, PvLIL proteins are significantly more hydrophobic and folded (*p* values are 4.3e−07 in both cases). Thus, our analysis suggests that PvLIL proteins lack typical features of PvLEA proteins and other members of the Group 3 LEA, namely their highly hydrophilic properties and the state of intrinsic disorder. These are important to LEA proteins because highly hydrophilic amino acid stretches provide their random-coil disordered state in aqueous solutions, which transforms into the α-helical conformation in a course of desiccation^14^.

The similarity of PvLIL proteins to PvLEA1 and PvLEA3 is restricted to the non-LEA regions.

**Figure 4:**
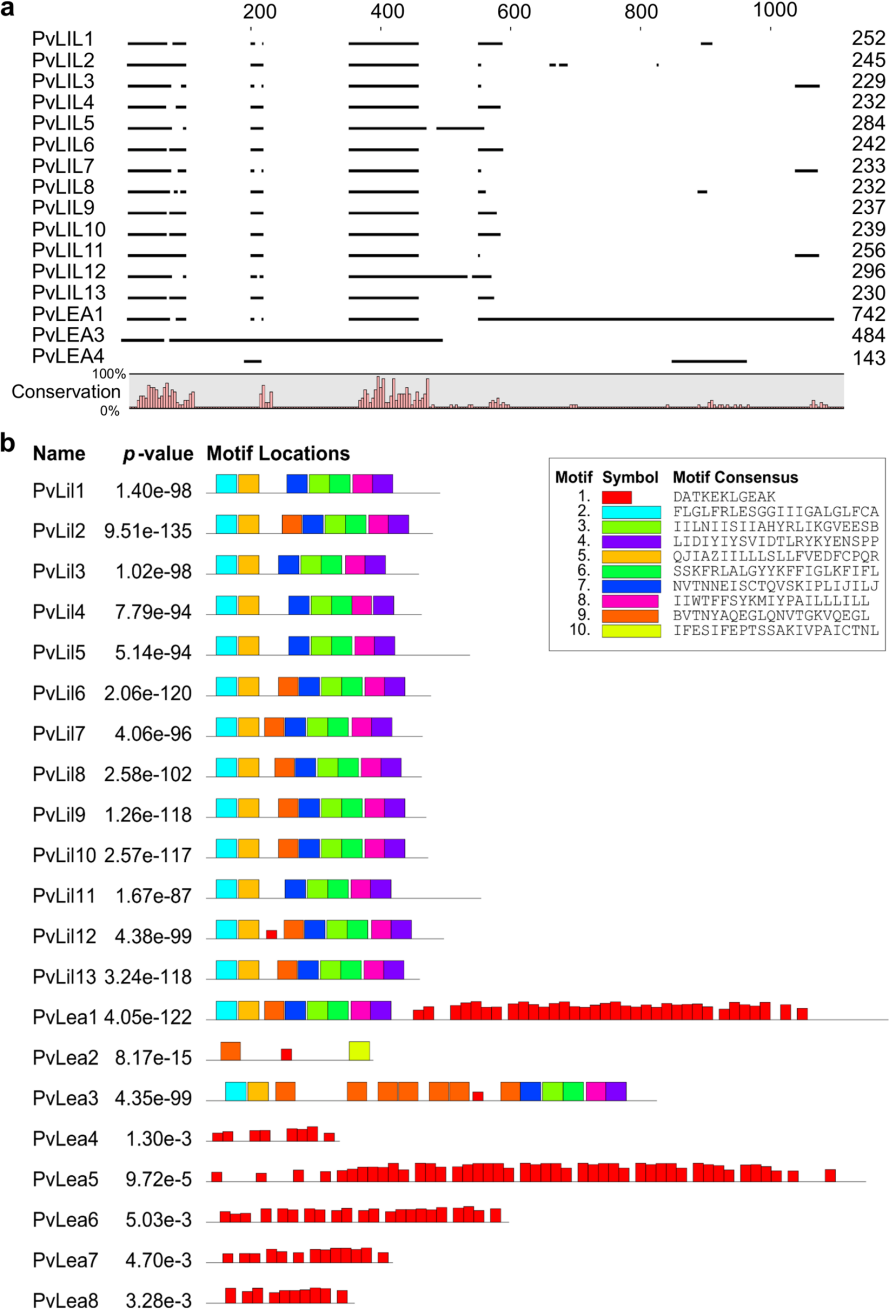
The similarity of PvLIL to PvLEA proteins and distribution of predicted motifs. (**a**) Multiple sequence alignment of 13 PvLIL and 3 PvLEA proteins. The similarity is restricted to N-terminus and C-terminus regions of PvLEA1 and PvLEA3, respectively. Alignment was performed in CLC Genomics Workbench 8.0 (QIAGEN, Denmark) with gap opening and extension penalties set to 10 and 0, respectively. (**b**) The amino acid motif composition of all PvLIL and PvLEA1–PvLEA8 proteins. The search for new amino acid motifs was performed on the MEME Suite server^20^ using classic search mode with any number of motif repetitions for all sequences of PvLIL and PvLEA proteins. We removed PvLEA9‒PvLEA27 from the bottom of the image for brevity. Maximum number of different motifs and maximum motif widths were set to 10 and 22, respectively. Found motifs are depicted as coloured blocks, whose height corresponds to their *p *value. Names of proteins and combined *p *value of all motifs for each protein sequence are given on the left. Removed proteins PvLEA9–PvLEA27 have one to four motifs of type #1 each with combined *p* values from 3.96e−22 to 1.19e−1, except for the PvLEA22 protein with no motif type #1 found.

To further investigate the relation between PvLIL and PvLEA proteins, we aligned PvLIL proteins with PvLEA1, PvLEA3 and PvLEA4, since the latter is a typical example of LEA proteins of Group 3^19^. The similarity of PvLEA1 and PvLEA3 to PvLIL proteins is limited to the regions near their N- and C-terminus, respectively (Fig. 4a). PvLEA4 protein is not aligned with PvLIL and located on alignment near the C-terminal region of PvLEA1 protein.

Then we studied the distribution of typical LEA protein features in the sequences of all PvLIL and PvLEA proteins using the MEME tool (https://meme-suite.org/)^20^. The specific tandemly repeated 11-mer motif “AKDXTKEKAXE” similar to a LEA_4 motif (PF02987) typical for the Group 3 LEA was found in sequences of PvLEA proteins, similarly to the previous findings of Hatanaka et al.^19^ (Fig. 4b, Table S2). This motif is absent in all PvLIL proteins except PvLIL12. Motifs detected in PvLIL proteins are not tandemly repeated and their amino acid composition is different to the previously described PvLEA protein motifs (Fig. 4b). These motifs are also present in PvLEA1 and PvLEA3 proteins and are absent in all other PvLEA proteins. In PvLEA1 protein, these motifs are located in PvLIL-similar region near the N-terminus, whereas in PvLEA3 proteins they are split between the C- and N-terminus. These regions of PvLEA1 and PvLEA3 also have a very low tendency toward disorder as suggested by IUPred2 and ANCHOR analysis (Fig. S1). Most of  PvLIL proteins lack any intrinsically disordered parts as predicted by IUPred2 and ANCHOR (Fig. S2). Only three of the studied proteins, PvLIL5, PvLIL8 and PvLIL11, have short regions with a disorder score exceeding the threshold for intrinsically disordered state. In all cases, such regions are located near the C-terminus of the protein (Fig. S2).

### PvLILs are targeted at the membrane and their conserved residues are located in transmembrane domains or nearby

**Figure 5:**
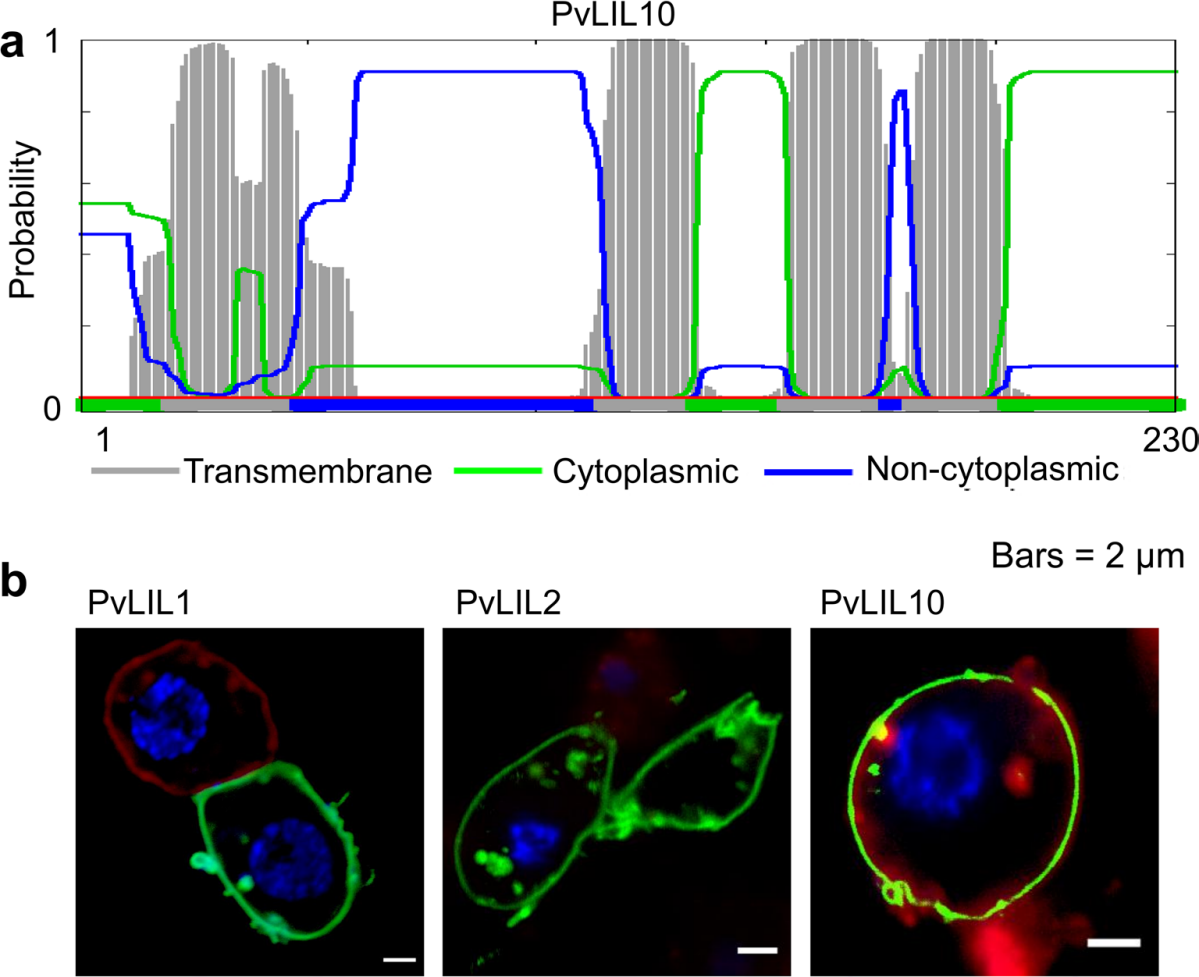
The representative transmembrane topology of PvLIL proteins (**a**) and actual localization of PvLIL-AcGFP chimeric proteins in cytoplasmic membrane of Pv11 cells (**b**). (**a**) Transmembrane topology for PvLIL10 protein sequence predicted by Phobius tool^54^ representing the typical topology of PvLIL proteins. Grey areas indicate the high probability of transmembrane allocation of corresponding sequence regions. The coloured lines indicate the different orientation of non-transmembrane loops: blue line for non-cytoplasmic and green line for cytoplasmic orientation probabilities. Red line indicates the absence of the signal peptide probability. (**b**) Localization of PvLIL1-AcGFP, PvLIL2-AcGFP and PvLIL10-AcGFP chimeras in the Pv11 cell line. Red staining: cell membrane labelling by CellVue Claret Far Red (Sigma, USA). Blue: DNA staining with Hoechst 33258.

**Figure 6:**
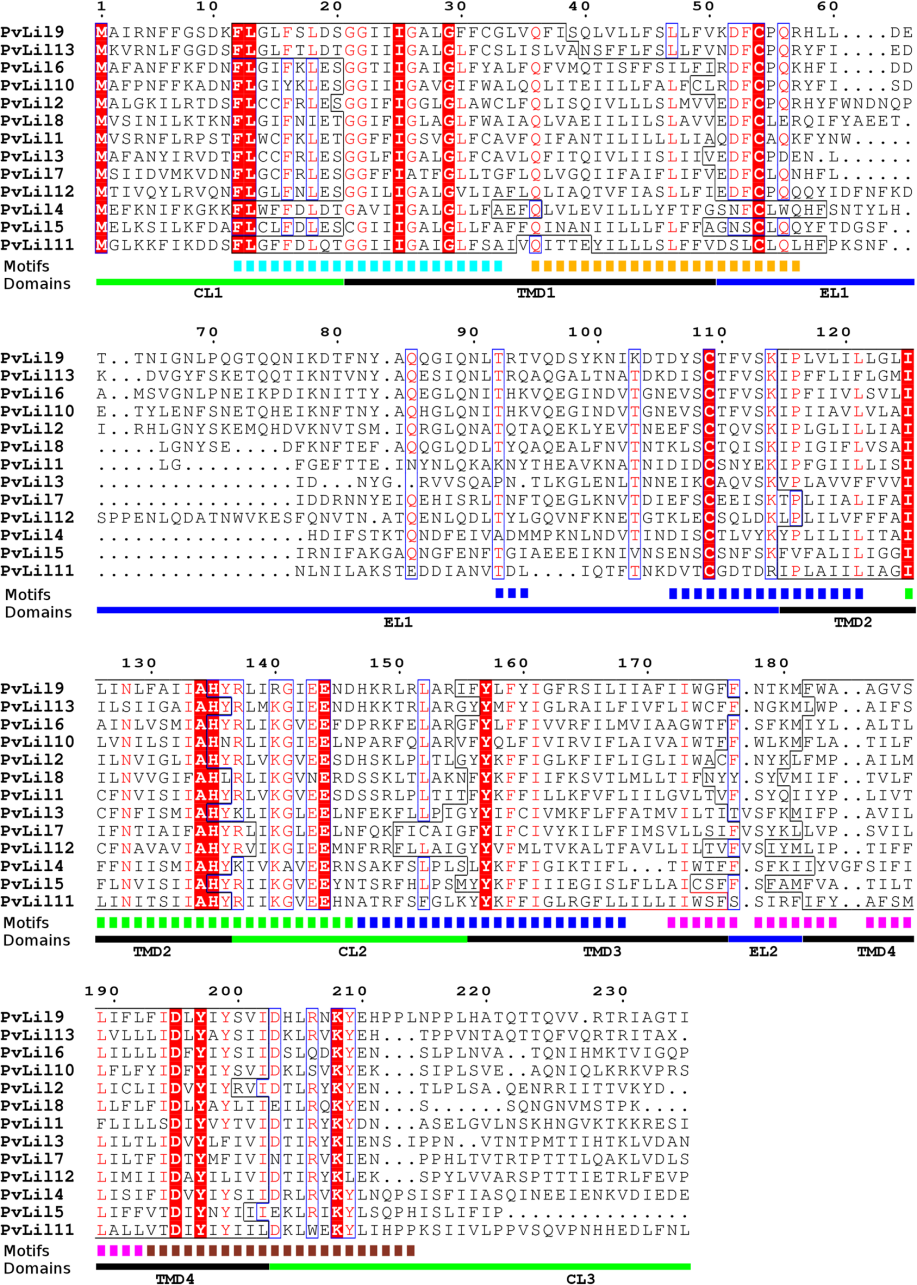
Distribution of domains and motifs across PvLIL sequences. *Red* highlighting and *red* coloured letters on alignment indicate amino acid residues with 100% and ≥ 70% conservation, respectively. Coloured dashed and solid lines below alignment depict PvLIL-specific motifs found by MEME. Solid lines below alignment indicate the position of domains predicted by TMpred: *black*—transmembrane domains (TMDs), *green*—cytoplasmic loops (CLs), *blue*—extracellular loops (ELs). The exact position of predicted transmembrane domains within each protein is denoted on alignment by thin black line. Figure prepared in ESPript 3.0^55^ from alignment obtained in CLC Genomics Workbench 8.0 (QIAGEN, Denmark). Annotation names were added in GIMP 3.0.

Previous data on PvLEA protein localization in the Chinese hamster ovary cell line or Pv11 cells revealed that most of them are located in the cytosol^19,21^. PvLEA1 was detected in the cell membrane and PvLEA3 in the endoplasmic reticulum or Golgi apparatus. Both proteins are predicted to possess transmembrane domains (TMDs) in their regions similar to PvLIL protein sequences by the Phobius tool (Fig. S1). All PvLIL proteins have four to five TMDs as predicted by the Phobius tool (Fig. 5, Fig. S2). Previously discovered PvLIL protein-specific motifs, which are present in all PvLILs, are also localized near these TMDs and are absent inside the extracytoplasmic loops (Fig. 6). PvLIL proteins have 15 fully conserved amino acid residues. These residues are mostly located in TMDs or nearby (up to nine residues apart, Fig. 6). The first extracellular loop is the most variable part of PvLIL sequences after the C-terminus region and it contains two cysteine residues that can form a disulfide bond.

We confirmed membrane targeting of PvLIL1, PvLIL2 and PvLIL10 proteins in vivo in Pv11 cells. Pv11 cells were transfected with vectors carrying PvLil genes in frame with AcGFP gene located downstream. These chimeras were placed under the control of the *P. vanderplanki*-derived 121 promoter, which ensures a high level of gene expression in a broad range of insect cell lines^22,23^. All three PvLIL proteins investigated produced chimeras localized in the plasma membrane of Pv11 cells, confirming membrane targeting of PvLIL proteins (Fig. 5b).

To investigate the structure of PvLil genes, we performed additional transcript prediction by StringTie using the RNA-seq data on larvae desiccation. Predicted transcripts were verified via cDNA cloning and Sanger sequencing. All PvLil genes are expressed in one splice form with the exception of PvLil4. PvLil4 is expressed in two splice forms, which differ due to the retention of 42 bp introns (Fig. S3). This retention introduces a stop codon downstream of the 81st nucleotide of the longest open reading frame (Supplementary Data 2). This ORF is functional as we can conclude from the fact that it encodes proteins with conserved residues (see below). The truncated ORF can be translated 204 bp downstream of this introduced stop codon from the double ATG codon, producing protein missing 81 amino acid residues at the N-terminus (Fig. S3). This constitutes nearly one-third of this protein, which typically contains 232 amino acids. Downstream of the introduced stop codon there are also three other potential ORFs, which are 87‒90 nucleotides long. They are probably unfunctional since they have no significant homology identified by TBLASTX search in the NCBI collection.

### PvLils are induced in anhydrobiosis and highly expressed in larval life cycle stage.

**Figure 7:**
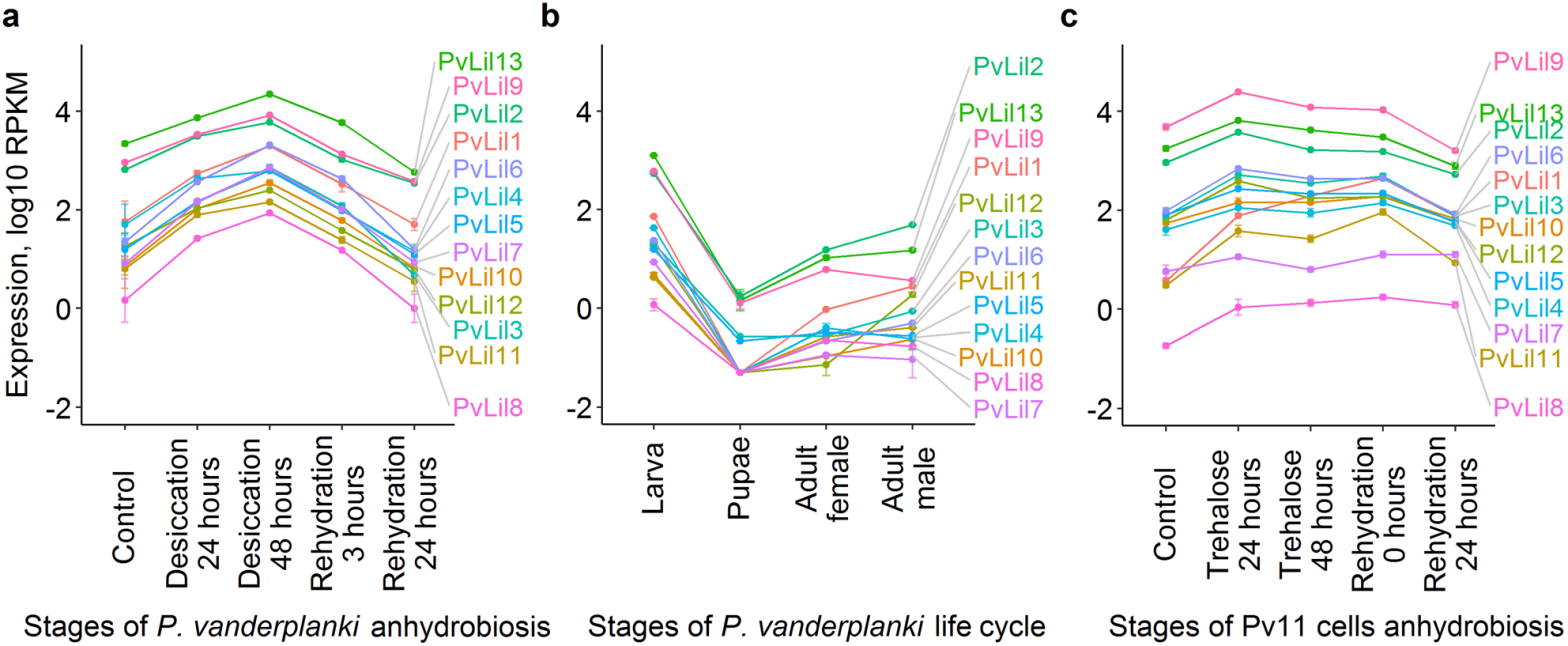
Expression of *PvLil* genes during *P. vanderplanki* larvae anhydrobiosis (**a**), at different stages of life cycle of *P. vanderplanki* (**b**) and during anhydrobiosis of Pv11 cells (**c**). Gene expression data were obtained via mapping of RNA-seq data on a merged assembly consisting of AUGUSTUS-based PvScaf 0.9 assembly with more accurate ARId annotation of ARId regions. Scaffolds in PvScaf 0.9 assembly matching sequences of ARId-located genes with 100% identity or *p* value less than 1e−60 in BLASTN program were removed to avoid ambiguous read mapping. Read mapping was performed with HISAT2 2-2.1.0^46^; counts and RPKM values were calculated using HTSeq 0.5.4p3 and edgeR 3.26.8, respectively^48,49^. Expression (log10 RPKM) is given on the y-axis, stages of desiccation or life cycle are on the x-axis of respective plots. Error bars depict standard deviation. Data for life stages contained zero expression values. These values were replaced with 0.05, which is a pseudocount equal to half of a minimum non-zero value. Thus we avoided the introduction of infinite values after logarithmic transformation.

Since *PvLil* genes were located together with *PvLea1* and *PvLea3* on the phylogenetic tree and are related to these genes, we speculated that they can also be associated with anhydrobiosis in *P. vanderplanki*. To study this association, we examined the expression profiles of *PvLil* genes during desiccation of the larvae and during its life cycle. In accordance with our published RNA-seq data set, all *PvLil* genes are induced in a course of desiccation (Fig. 7a). In addition to this association, during life cycle of *P. vanderplanki PvLil* genes also have the highest expression in a larval stage, which is the only stage when the midge is able to survive desiccation^24^ (Fig. 7b). Notably, the expression levels of the investigated genes in larvae 24 h post-rehydration are lower than in the control. Such an abrupt expression decrease may be interpreted as if *PvLil-*encoded protein products are necessary for the preparation of cells of *P. vanderplanki* for the water loss and are not needed for the subsequent post-rehydration recovery. We found a similar pattern of *PvLil* expression in our recently produced data set that covers anhydrobiosis induction in *P. vanderplanki-*derived Pv11 cell culture (Fig. 7c)^21^. All *PvLil* genes were induced during anhydrobiosis onset in Pv11 cells, which are the only one known animal cell culture model of anhydrobiosis. In this case, anhydrobiosis induction consists of the incubation of Pv11 cells in trehalose for 48 h, followed by their desiccation. All *PvLil* genes except *PvLil7* and *PvLil8* had a clear expression peak during trehalose treatment (Fig. 7c). Thus, *PvLil* genes are associated with anhydrobiosis in both larvae of *P. vanderplanki* and the Pv11 cells as a model of anhydrobiosis.

## Discussion

In this study, we investigated a new group of *PvLil* genes that are localized together with genes of LEA proteins in the *P. vanderplanki* genome. Thirteen *PvLil* genes are located with 20 *PvLea* and five other genes in a nearly 100 kilobase genomic region, forming tandem groups consisting of two to four *PvLil* genes each. Such an expansion of paralogous genes within one genomic region is typical of the *P. vanderplanki* genome^17^. Nine regions in the *P. vanderplanki* genome populated by such paralogous genes were named ARIds (anhydrobiosis-related gene islands) because of both high induction and high expression of incorporated genes in anhydrobiosis^17^. It was concluded that ARIds themselves and the genes included are important for anhydrobiosis through a “guilt-by-association” approach. The location of genes of well-known effectors of anhydrobiosis within ARIds provides further evidence of this connection. LEA proteins of Group 3, whose genes are co-located with *PvLil* genes, are extensively studied in a wide variety of organisms, demonstrating both the association with water deficit and experimentally verified effects, such as reducing surface-induced aggregation of proteins, protection and stabilizing the lipid bilayers and mitochondria, increasing cytoplasmic conductivity of cells, reinforcing sugar glasses^9,25–28^. The latter include the transition from an intrinsically disordered to a structured state on dehydration, the reinforcement of trehalose-based glassy matrix and direct protection of proteins against desiccation-induced aggregation demonstrated for PvLEA22 protein^14,15^. Similar effects were demonstrated for PvLEA4 proteins and many others^16^, reviewed in^26,29^. These actions are important for anhydrobiotic organisms since dehydration often causes protein aggregation. The formation and reinforcement of biological glass within the cells is linked to anti-aggregation, since it can mechanically prevent denaturation and mechanical disruption of cellular components^29^.

*PvLil* genes form a clade within *PvLea* genes on the phylogenetic tree. Most close homologues of *PvLil* genes are *PvLea1* and *PvLea3*, which are located together with six *PvLil* genes in *P.vanderplanki* genome, forming tandem groups with four and two *PvLil* genes, respectively. Taken together with the sequence similarity to *PvLil*, this location of *PvLea1* and *PvLea3* genes suggests a common origin of these genes with *PvLil* genes by gene duplication and divergence. These processes are considered the major pathways of the new gene’s appearance^30,31^. The presence of highly expanded families of diverged paralogous genes within ARId regions is typical of the *P. vanderplanki* genome^17^. Many of these paralogous genes become induced during desiccation and encode proteins whose function apparently can be useful for surviving desiccation. For example, such families include thioredoxins, which can alleviate the huge oxidative damage that occurs during water loss^3^, and protein repair methyltransferases, which could be implicated in the reparation of proteins damaged during desiccation^17^. Taken together with the absence of these paralogous genes in the genome of the congeneric midge *P. nubifer*, which is unable to survive desiccation, these data suggest that multiple gene duplication exemplified by *PvLil* genes should be an important mechanism of the evolution of the *P. vanderplanki* genome related to its anhydrobiotic phenotype. The presence of PvLIL-like parts containing TMDs in PvLEA1 and PvLEA3 suggests a gene fusion between the proto *PvLil* gene and some of the *PvLea* gene’s copy in the past. However, the determination of the exact phylogenetic history of these genes and order of their appearance is beyond the scope of our present work. None of PvLEA1 and PvLEA3 homologs, which similarity expands on PvLIL-like areas of these proteins, has transmembrane domains or conserved aminoacids of PvLIL.

PvLIL proteins have significantly lower hydrophilicity and predicted intrinsic disorder score, and do not bear motifs typical of PvLEA proteins. Thus, even though PvLIL proteins are related to PvLEA1 and PvLEA3, they are not members of the Group 3 LEA proteins. Moreover, PvLEA1 and PvLEA3 themselves are not typical PvLEA proteins because of the presence of regions similar to PvLIL, which are also not in a disordered state and are probably transmembrane. These data are in accordance with previous findings that PvLEA1 and PvLEA3 proteins incorporate specific motifs in their N- and C-terminus regions that are not similar to motifs of other PvLEA proteins^19^. However, they also bear LEA-like regions with a higher disorder score and LEA-like motifs and do not aggregate and precipitate even after boiling, similarly to typical LEA proteins^32^. This combination ensures that the localization of PvLEA1 and PvLEA3 proteins in the cell membrane may be related to protection of the membrane against the fusions that occur during desiccation^33^. Since PvLEA3 protein is located in the endoplasmic reticulum or Golgi apparatus, its effect is also related to the membrane of the corresponding subcellular compartment rather than the cell membrane^19,21^.

Since PvLILs do not possess any known protein domains, we currently cannot decipher their exact function. However, the presence of four or five predicted TMDs in each PvLIL suggests that these proteins do indeed pass through the membrane in contrast to proteins with one domain that may be just anchored in the membrane^34^. This structure is similar, for example, to the structure of innexins, having four TMDs with two extracellular and one intracellular loop between the transmembrane helix^35^. Innexins are transmembrane proteins forming gap junction channels in cells of different invertebrates including *Caenorhabditis elegans* and *Drosophila melanogaster*.

Because PvLEA3 was observed in the endoplasmic reticulum or Golgi apparatus, the function of some PvLIL proteins can also be related to the membrane of the corresponding compartment rather than the cell membrane. Some glimpses of PvLIL function can be derived from conservative amino acid residues within PvLIL sequences. First, the location of two conserved cysteine residues on the edges between the first extra cytoplasmic loop and the TMD suggests the importance of this loop for the PvLIL function. Disulfide bonds between conserved cysteine residues in extracellular loops are shown to be important for the function of a number of transmembrane proteins. These include cell adhesion molecules such as innexins and connexins^36,37^ and receptors acting via G-coupling^38,39^ or as ion channels^40^. The formation of such disulfide bonds affects the regulation of receptor protein activity via stabilization of the extracellular loop, ensuring its correct conformation. Such cysteine residues are also involved in the trafficking of a channel protein to the cell surface^40^. This example is especially important since channel proteins are important for anhydrobiosis of *P. vanderplanki*, as was shown for one of its aquaporins, *PvAqp1*^41^. This gene is induced in response to desiccation and is involved in water removal during the induction of anhydrobiosis. Since desiccation causes increased concentration of all kinds of molecules in both extracellular fluid and inside cells of *P. vanderplanki*, transport related to this concentration jump is a plausible hypothesis for PvLIL’s function. Observed variation both in the sequence and length of PvLIL extracellular loops may be linked to the difference in their specialization. Interestingly, the structure of PvLEA3 and PvLEA1 is similar and 11 out of 15 amino acid residues fully conserved in PvLIL are also conserved in these proteins, including two cysteine residues in the first extracellular loop. A whole LEA-like part of PvLEA3 is located within its first extracellular loop.

As we revealed, the *PvLil4* gene has two different splice forms whose existence we confirmed by cDNA cloning. Previously the presence of alternative transcripts was observed for up to 53% of *P. vanderplanki* genes^42^. These genes include glutathione peroxidase, present in four splice forms and the heat shock factor (HSF)^17,42^. The most prominent difference in *Hsf* transcripts originates from the retention of the fifth exon and adjacent introns, which is close to the observed difference in *PvLil4* transcripts. Among all alternatively spliced genes, 338 have exons with significant changes in the splicing pattern that occurs during anhydrobiosis^42^. An interesting feature of *PvLil4* is the introduction of the stop codon near the 5′ end of the transcript, which probably results in the production of truncated protein and has not been reported for *P. vanderplanki* before.

The expression patterns of *PvLil* genes during the desiccation process in the *P. vanderplanki* larvae and during the trehalose treatment in the Pv11 cell line and the high expression rates at the larval stage during the life cycle might display their connection with the anhydrobiosis process. The difference in the expression profiles throughout the dehydration procedure in the Pv11 cells and in the larvae might be a reflection of different needs in the quantity of translated PvLIL proteins in the whole organism (the larva) and in the single cells, derived from *P. vanderplanki* egg masses. It depends on the function of PvLIL proteins in the anhydrobiotic process and their possible expression specificity in some tissues or organs. The lower expression levels after rehydration of the larvae and the cell culture in comparing the control samples indicate that PvLIL proteins might not be involved in the reparation process.

The discovered high rates of expression of the PvLIL proteins during the desiccation process and the lack of relevant homologues in the NCBI database may indicate the emerging of the new group of proteins, contributing to the anhydrobiosis process. The chironomid *P. vanderplanki* is nowadays the most complex known anhydrobiotic species. The complexity of the organism could lead to the new protein-coding genes originating to facilitate the connection of anhydrobiosis machinery in the different events during the dehydration process. The PvLIL proteins are quite different in their amino acid sequence properties and the expression patterns, despite their similar topology and length. The divergence inside the relatively small group of proteins should be evidence of their evolution process. The gene duplication process is followed by either degradation of the new copies or accumulation of the new mutation in the new genes’ sequences, allowing them to acquire a new function or to carry out only part of the ancestor’s function. The partial gene duplication should also be the origin of the new gene group, if the duplicated part of the gene contained the important domains, and it can give rise to the structural and functional complexity of the newly emerged gene^43,44^. However, we cannot be sure of the evolutionary occurrence of the *PvLil* genes without the observed predecessor gene in the *P. vanderplanki* genome, which led to a new gene group.

## Materials and methods

### Cell line

The Pv11 cell line isolated from the egg masses of *P. vanderplanki* was cultivated following previously published protocols^45^. Briefly, we cultivated Pv11 cells in IPL-41 medium (Gibco, USA), supplemented with 10% foetal bovine serum (HyClone, USA) and 2.6 g/l tryptose phosphate broth, in a non-humidified incubator at 25 °C.

### Gene cloning

ARId Sequences of PvLil genes, the genome assembly of *P. vanderplanki* (version PvScaf 0.91) and RNA-seq data were downloaded from the MidgeBase database at https://bertone.nises-f.affrc.go.jp/midgebase/. The StringTie assembler was used to predict unannotated versions of PvLil genes using default settings and combined replicates of RNA-seq for each type of sample. Primers for cloning of coding sequences of all PvLil transcripts were designed with Primer3Plus 2.4.2; their sequence is described in Supplementary Table S1. RNA was isolated from dried *P. vanderplanki* larvae using TRIzol reagent (Thermo Fisher Scientific, USA) and synthesized cDNA with an iScript Select cDNA Synthesis Kit (Bio-Rad, USA) in accordance with the manufacturer’s protocols. We amplified CDS of 13 PvLil genes from the obtained cDNA with Q5 High-Fidelity DNA Polymerase (New England Biolabs, USA) following the standard protocols. The resulting DNA fragments were cloned using HindIII and XbaI restriction enzymes and T4 DNA Ligase (Roche Applied Science, Germany) into the vector containing 121 promoter. This is a novel promoter derived from *P. vanderplanki* that ensures strong expression of genes in cells of *P. vanderplanki* and other insect cell lines^22,23^. Cloned fragments were sequenced by Sanger using the equipment of the Interdisciplinary Centre for shared use of Kazan Federal University. Nucleotide sequences of coding regions of PvLil genes are shown Supplementary Data 1.

### Subcellular localization of PvLEA-GFP fusion proteins in Pv11 cells

Pv11 cells were transfected with NEPA21 Super Electroporator (NEPA GENE, Japan). We cultivated Pv11 cells for 24 h after transfection in standard conditions. After cultivation, transfected Pv11 cells were stained with CellVue Claret Far Red (Sigma, USA) and Hoechst 33258 (Sigma, USA) for visualizing cell membranes and DNA, respectively. We examined the localization of fused PvLIL-AcGFP1 proteins in cell culture using an LSM 780 laser confocal microscope (ZEISS, Germany) in the Interdisciplinary Center for Analytical Microscopy of Kazan Federal University.

### RNA-seq

We used published RNA-seq data sets to quantify *PvLil* expression in Pv11 cells, in larvae of *P. vanderplanki* during their desiccation and in different life cycle stages of *P. vanderplanki*^19,21^. Briefly, Pv11 cells were subjected to the established procedure of anhydrobiosis induction consisting of treatment with trehalose for 48 h and drying of Pv11 cells for 10 days in a desiccator, followed by rehydration and cultivation^21^. *P. vanderplanki* culture was reared and larvae were slowly dried in accordance with the previously published protocol^24^. The produced RNA-seq data sets were mapped onto *P. vanderplanki* genome (version 0.9) and AUGUSTUS-produced gene annotations (version 0.91) merged with sequences and annotations of Anhydrobiosis-related gene Island (ARId) to obtain better accuracy of *PvLil* gene quantification^21^. RNA-seq data were processed via mapping by HISAT2 version 2-2.1.0^46^, sorting of resulting SAM files by SAMtools 1.9-52^47^, obtaining corresponding counts using HTSeq 0.5.4p3^48^ and calculation of RPKM values using edgeR 3.26.8^49^.

### In silico analysis

All *PvLil* gene sequences were taken from a MidgeBase genome browser (https://bertone.nises-f.affrc.go.jp/midgebase/). We performed a search of the possible orthologues of *PvLil* genes in the NCBI database using the Basic Local Alignment Search Tool (BLAST) (https://blast.ncbi.nlm.nih.gov/Blast.cgi) with a standard set of parameters for BLASTN and discontiguous MegaBLAST algorithms. The multiple nucleotide sequences alignment of *PvLil1-13* and *PvLea1-27* was performed by CLC Genomics Workbench 8.0 (QIAGEN, Denmark). The phylogenetic tree was constructed by using the Maximum Likelihood method based on the General Time Reversible model in MEGA7^50^. The tree with the highest log likelihood was chosen as the consensus tree and edited in MEGA7. The initial tree for the heuristic search was obtained by applying the Neighbor-Joining method to a matrix of pairwise distances estimated using the Maximum Composite Likelihood (MCL) approach. The analysis involved 40 nucleotide sequences. There were a total of 2,229 positions in the final dataset. To search the known protein family’s motifs from Pfam databases we scanned the PvLIL amino acid sequence using the HMMER tool (https://www.ebi.ac.uk/Tools/hmmer/)^51^. We set parameters for the significant E-value for the sequence as 0.01 and performed our search throughout the PDB database. The amino acid sequences encoded by *PvLil* genes were examined using the ProtParam tool (https://web.expasy.org/protparam/)^18^ for molecular weight, theoretical isoelectric point (pI) and grand average of hydropathy (GRAVY) prediction. The unfoldability of the PvLIL proteins was calculated by FoldIndex (https://fold.weizmann.ac.il/fldbin/findex)^52^. We computed potential intrinsically disordered regions in the PvLIL protein sequences and their possible binding sites, we downloaded amino acid sequences into the IUPred2a tool (https://iupred2a.elte.hu/) and chose the long disorder type of predictions^53^. A two-sided Wilcoxon test was implemented to compare properties of PvLIL and PvLEA proteins using the built-in algorithm of ggpubr R package version 0.2.3 (https://github.com/kassambara/ggpubr). Continuity correction was introduced to compensate for the presence of tied values; n was 13 and 27 for PvLIL and PvLEA, respectively. In addition, for each protein characteristic, we performed 1,000 replicates of the two-sided Wilcoxon test using the R stats package version 3.5.3. Each iteration we introduced normally distributed noise (± 1 × 10^–3^ of a maximal value for a given characteristic) to get rid of tied values. The obtained *p* values were in all cases lower than the *p* values calculated in the test with correction. Thus, higher *p* values obtained in the test with correction were used. Data for PvLEA proteins were taken from^19^. They were reproduced to ensure consistency with our analysis of PvLIL proteins. The transmembrane domains in the PvLIL protein sequences were predicted with the Phobius tool, using normal prediction mode (https://phobius.sbc.su.se/)^54^. The amino acid motif search was performed by using the MEME online program (https://meme-suite.org/)^20^ for identifying the conservative LEA_4 motif or finding some new repetitive amino acid sequences in PvLEA and PvLIL proteins. The parameters were set as follows: classic mode, maximum number of different motifs 10; maximum motif widths 22 and any number of motif repetitions.

## Conclusions

Our data provide the first insights into amino acid sequence properties and the expression patterns of the new gene group *PvLil* in *P. vanderplanki*. PvLIL proteins share some similarity with two members of the PvLEA protein group; however, they do not possess the essential features of the LEA group, such as repetition of the motifs and a high disorder score. PvLILs contain four or five transmembrane domains each, with most conservative amino acid residues being located in them or nearby. All PvLIL-encoding genes are induced in response to anhydrobiosis both in the *P. vanderplanki* larvae and in the *P. vanderplanki*-derived cell line Pv11.

## Supplementary information

Supplementary Information.
