# Supplementary material for: New group of transmembrane proteins associated with desiccation tolerance in the anhydrobiotic midge Polypedilum vanderplanki
Source: Sci Rep. 2020 Jul 15;10:11633. doi: 10.1038/s41598-020-68330-6 (PMC7363813; doi:10.1038/s41598-020-68330-6)
Supplement: Supplementary file 1 — Supplementary Information. [file 41598_2020_68330_MOESM1_ESM.pdf]

New group of transmembrane proteins associated with desiccation tolerance in the anhydrobiotic midge *Polypedilum vanderplanki*

Taisiya A. Voronina[1*¶], Alexander A. Nesmelov[1¶], Sabina A. Kondratyeva[1], Ruslan M. Deviatiiarov[1], Yugo Miyata[2], Shoko Tokumoto[3], Richard Cornette[2], Oleg A. Gusev[1,4], Takahiro Kikawada[2,3*], Elena I. Shagimardanova[1*]

[1] Institute of Fundamental Medicine and Biology, Kazan Federal University, Kazan, Russia

[2] Division of Biotechnology, National Institute of Agriculture and Food Research Organization, Tsukuba, Japan

[3] Graduate School of Frontier Sciences, The University of Tokyo, Tokyo, Japan

[4] KFU-RIKEN Translational Genomics Unit, RIKEN, Yokohama, Japan

¶ These authors contributed equally to this work.

* Corresponding authors

Email: vorotaisiya@gmail.com
kikawada@affrc.go.jp
rjuka@mail.ru

Gene	Primer sequences (5' - 3')	
	**Forward primer**	**Reverse primer**
PvLil1	cgcgaagctttgatggtttcaagaaattttttaagacca	ccgtctagaaactttttcttttgcttcaaca
PvLil2	cgcgaagctttgatggcattaggaaaaatttttttgaga	ccctctagactttgttgttaatttttctttagcgt
PvLil3	cgcgaagctttgatggcttttgcaaattatatcagagt	ccgtctagatacatatactttaggaattgt
PvLil4	gcgggatccaagctttgatggaatttaagaatatctttaaaggg	ccgtctagaaaaatgtaacaaaaatccagtt
PvLil5	cgcgaagctttgatggaactaaaaagtatatt	ccgtctagaattaacacaaacaacttcatca
PvLil6	cgcgaagctttgatggcatttgcaaatttctt	cgctctagaataatttacatgatattgattccgtga
PvLil7	cgcgaagctttgatgagtataatagatgttatgaaagt	ggctctagattctggtacaataagtctgactgga
PvLil8	gcgggatccaagctttgatggtatcaatcaatattttaaaaacc	ccgtctagatttgaactgcttggggtgct
PvLil9	cgcgaagctttgatggcaat cagaaactttttggt	cgcctctagactcatcaatagttccagcaattct
PvLil10	cgcgaagctttgatggcatttccaaatttttcaaagc	cgctctagaaatttgaactc tttctggtaaatcaga
PvLil11	cgcgaagctttgatgggattaaaaaagttcattaaagac	ggctctagaagttgaaattgagattttatctttct
PvLil12	cgcgaagctttgatgacaatagtacaatatttacgagt	ccgtctagaatccaataattttggtataataattct
PvLil13	cgcgaagctttgatgaaggtcagaaatttatttggaggc	ggtctagatcaagcagtgattcttgttcgttg

Supplementary Table S1. The nucleotide sequences of *PvLil* coding sequences cloning primers

The motif ID	Regular Expression	Sequence logo
**1**	D[AT]T[KVA][ED]KLG[ED]AK	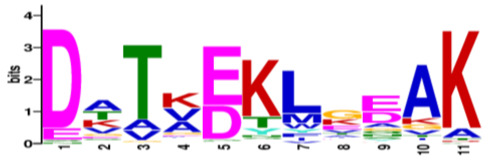
**2**	FL[GC][CLI]F[RDK]L[ED][ST]GGI[FI]IGA[LV]GL[FL][CW]A	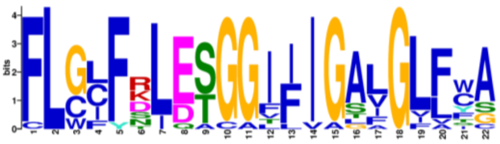
**3**	I[ICL][FLI]N[IVL][IV][SAG][IM]IAHYR[LI][IV]KG[VI]EE[SL][ND]	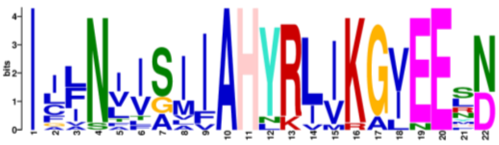
**4**	[LF]ID[IVL]YI[YF][IS][VI]ID[TK][LI]R[VY]KY[EL]N[ST]P[PH]	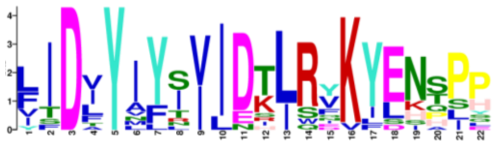
**5**	Q[LIF][IVF][AT][QEN][IT][IV][LF][LI][LFI]SL[LF]FVE[DN]FC[PL]Q[RQ]	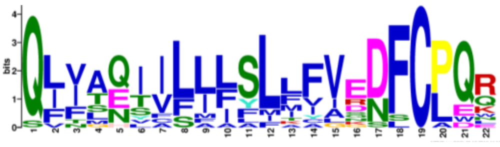
**6**	[FHS]S[KR][FL][PR]L[APT][LIR]G[YF]YKFFI[GV][ILF][KR]F[ILV][FL][LF]	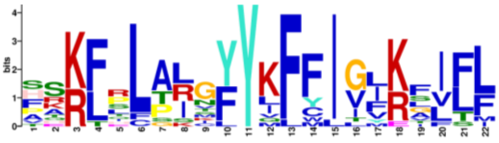
**7**	[ND]VT[NDG][NT][ED][ILV][ST]C[TS][QF][VI]SKIP[LF][GIA][LI][IV][LF][IL]	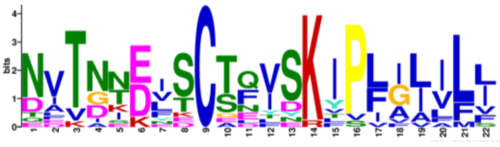
**8**	[IAG][ILV]W[TS]F[FV][SN][FY]KM[IFL][YW][PA][AT][IV][LSV][LFT]L[IL][LF]L	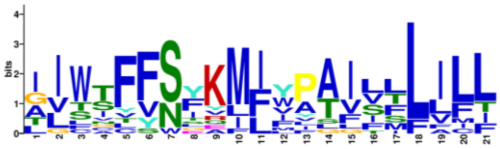
**9**	[DN]VT[GN]Y[AI][QK]E[GN][LI]Q[ND][VL][TA][GN][KT][VA]Q[ED][GK][LFI]	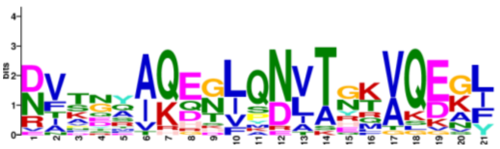
**10**	IFE[SG][IV]FEPT[ST]SAKIVP[AV][IV]CTNL	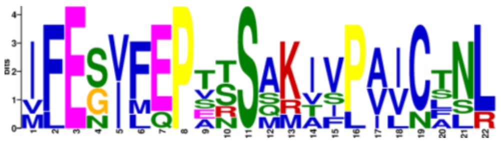

Supplementary Table S2. The consensus motifs identified in MEME analysis of PvLIL and PvLEA protein sequences. Motifs were found in the MEME Suite program. Motif 1 and Motif 10 are more specific for PvLEA proteins, whereas Motifs 2–8 were found only in 13 PvLIL proteins, PvLEA1 and PvLEA3. Motif 9 was found in 8 out of 13 PvLIL proteins and in PvLEA1, PvLEA2, PvLEA3 and PvLEA22.

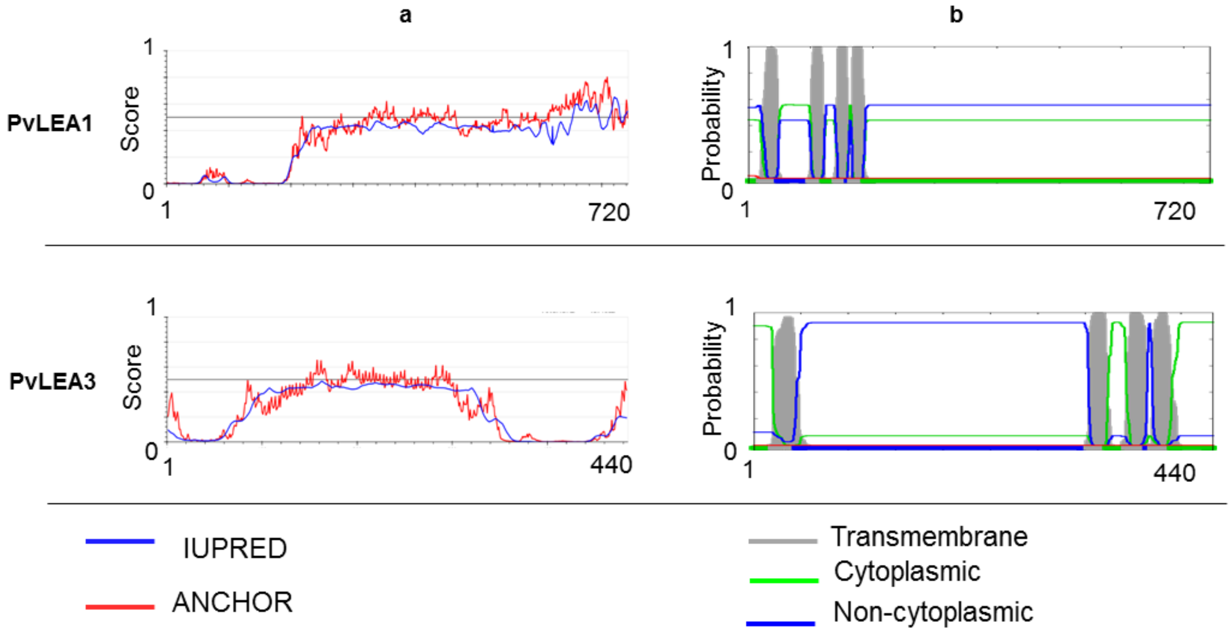

Supplementary Figure S1. Predicted disordered protein regions (**a**) and the transmembrane topology (**b**) in PvLEA1 and PvLEA3 proteins. High values of ANCHOR2 and IUPred2 scores indicate a disordered state. Regions of similarity to PvLIL proteins are limited to the N-terminus region in the case of PvLEA1 and the N-terminus and C-terminus regions for PvLEA3. *Blue* lines show the IUPred score, *red* lines show the ANCHOR score. Both PvLEA1 and PvLEA3 are predicted to possess four transmembrane domains, located in regions similar to PvLIL proteins.

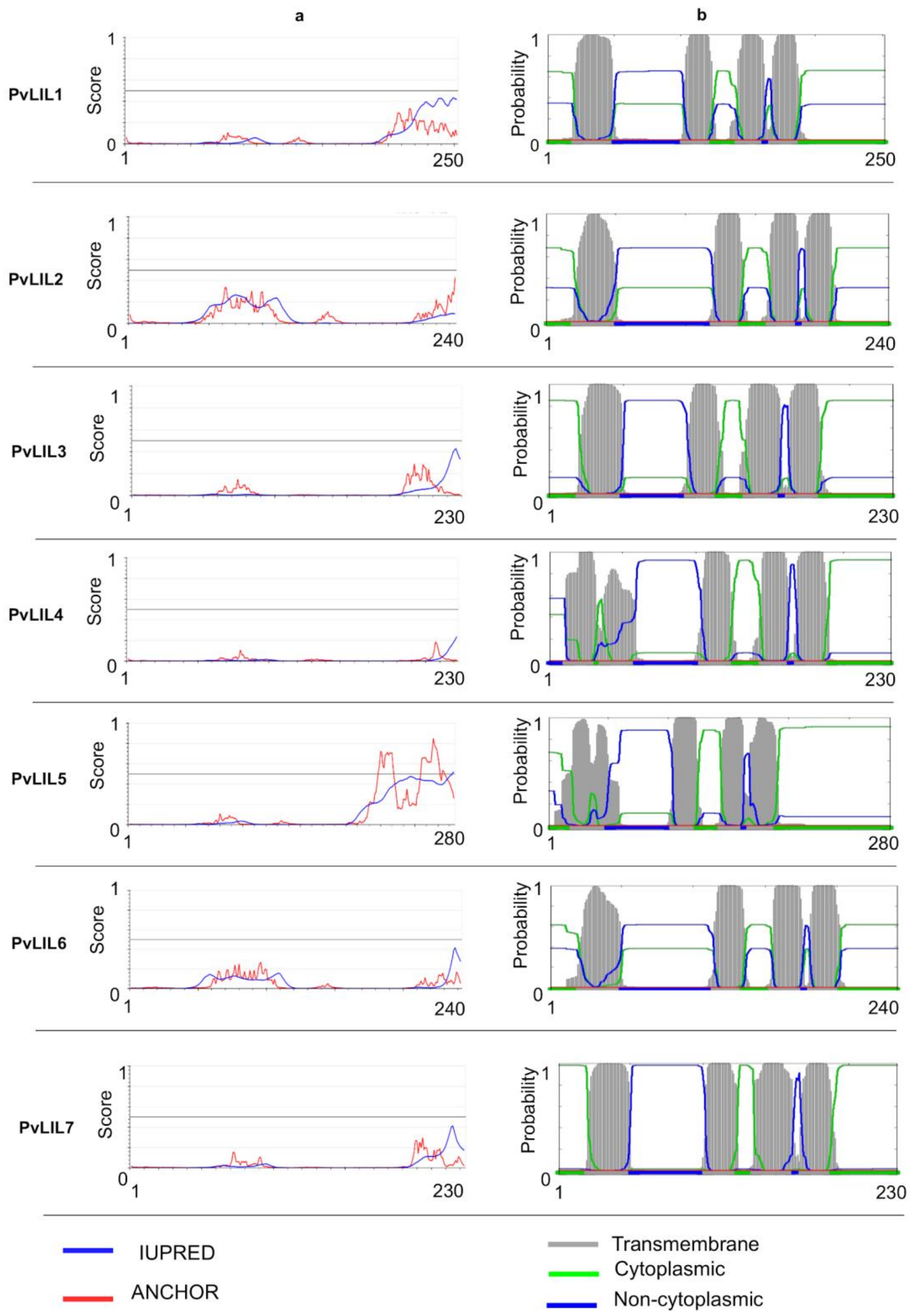

Supplementary Figure S2. Predicted disordered protein regions (**a**) and the transmembrane topology (**b**) in PvLIL proteins. All PvLIL proteins are predicted to obtain from four to five transmembrane domains with a relatively long extracytoplasmic loop in between the first and second transmembrane domains, and cytoplasmic-orientated C-terminus. Only for PvLIL5, PvLIL8 and PvLIL11 did the disorder score exceed the threshold in the C-terminus regions. **a**: *blue* lines show the IUPRED score, *red* lines show the ANCHOR score. **b**: *grey* blocks depict the transmembrane domains, *blue* lines the extracytoplasmic orientation and *green* lines the cytoplasmic orientation of protein regions.

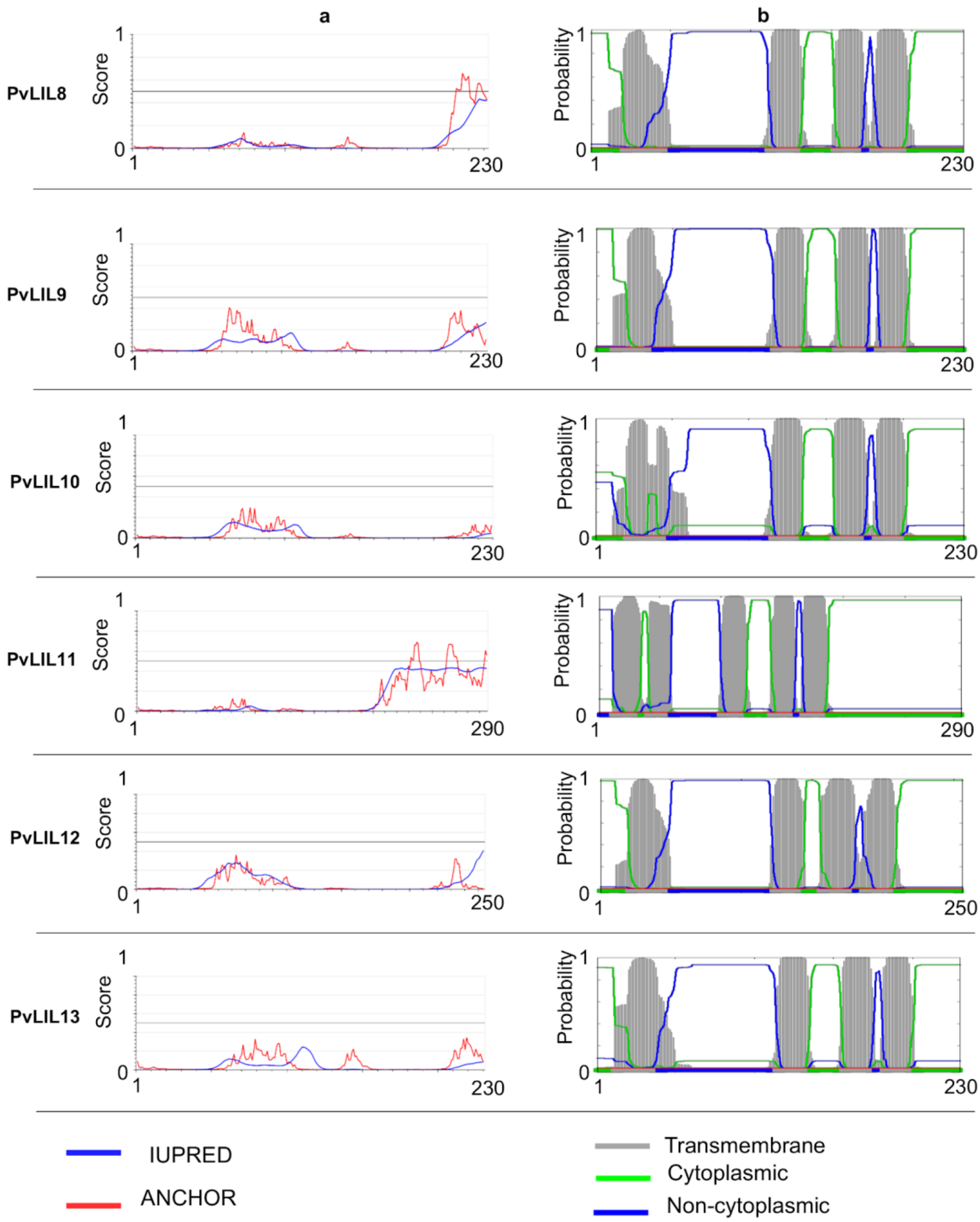

Supplementary Figure S2. (continued)

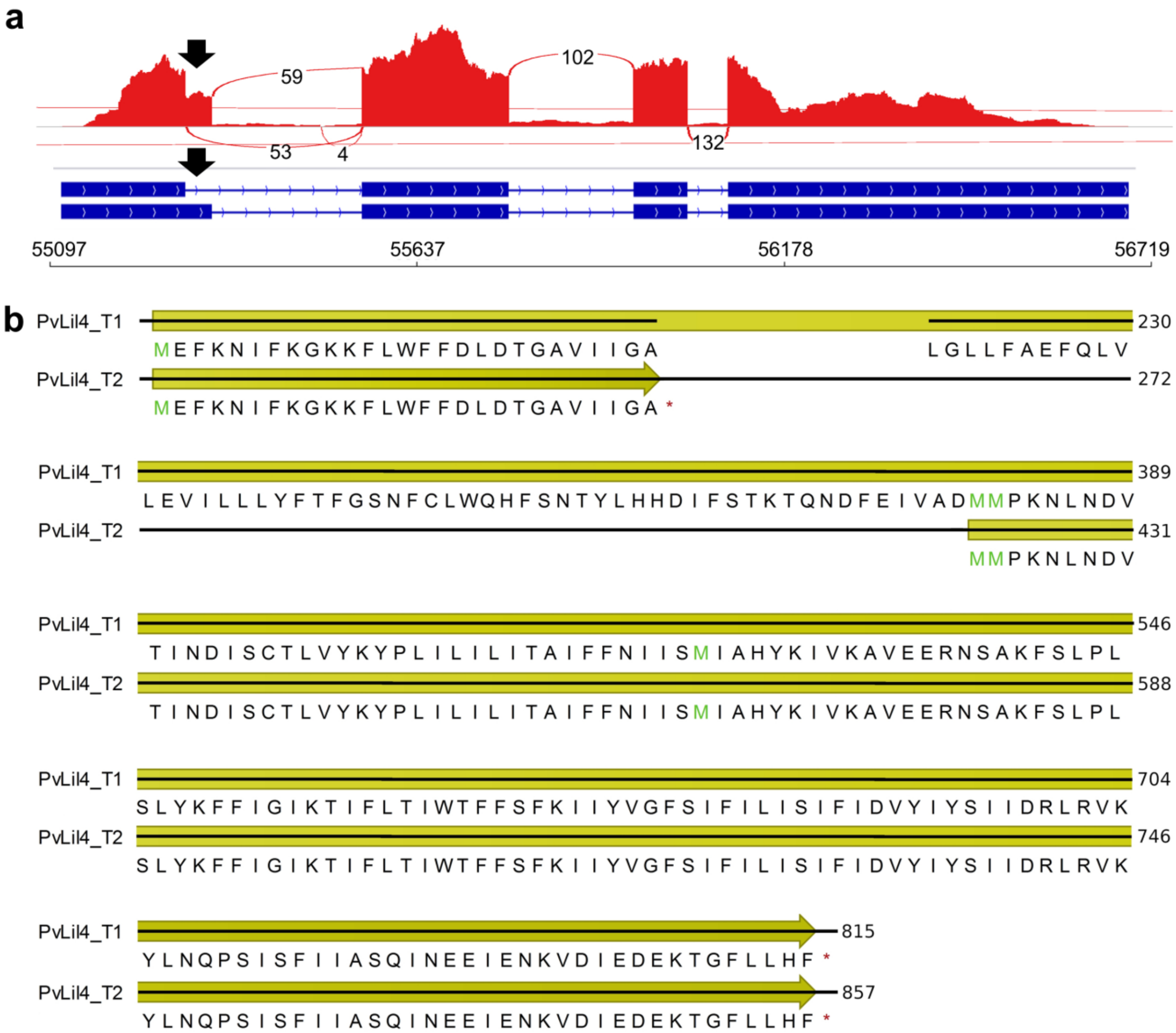

Supplementary Figure S3. Structure of *PvLil4* splice forms. **(a)** Sashimi plot and schematic of *PvLil4* splice forms in RNA-seq for control *P. vanderplanki* larvae, predicted by StringTie software. Retained intron is indicated by black arrow. Structure of splice forms predicted in other stages is identical to the presented one. Numbers indicate quantity of junctions detected in whole control larvae RNA-seq with two replicates combined. (**b**) Structure of ORFs in *PvLil4* splice forms. Splice forms are indicated on the left side, ORFs are depicted as yellow arrows, black line represents nucleotide chain. Translation to proteins is given below each transcript.

Supplementary Data 1

Nucleotide sequences of coding regions of *PvLil* genes in FASTA format

>PvLil1
ATGGTTTCAAGAAATTTTTTAAGACCAAGCACTTTTTTGTGGTGTTTTAAGTTGGAAACTGGT
GGATTTTTTATTGGCTCTGTTGGACTTTTTTGCGCAGTTTTTCAAATTTTTGCCAACACAATTT
TAATCTTATTGCTTTTAATTGCTCAAGACTTTTGTGCTCAAAAATTTTATAATTGGCTTGGATT
TGGTGAATTTACAACTGAAATAAATTACAATTTACAAAAAGCAAAAAATTATACACATGAA
GCTGTAAAAAATGCTACAAACATTGACATTGATTGCTCAAATTATGAAAAAATTCCTTTCGG
AATAATTTTGCTGATTTCAATTTGCTTCAATGTAATTTCCATTATTGCACACTACCGTCTTGTC
AAAGGAGTTGAAGAGTCTGATTCATCAAGATTGCCATTGACCATTACATTTTATAAATTTTTC
ATTTTATTGAAATTTGTGTTTTTGATTATTTTGGGAGTTTTGACAGTTTTTTCATATCAAATTA
TTTATCCATTAATCGTAACTTTCTTGATCCTTTTGAGTGATCTTTATGTTTACACAGTCATTGA
CACAATTCGCTATAAATATGATAATGCTTCAGAATTGGGAGTTTTAAACTCTAAACATAATG
GTGTTAAGACTAAGAAACGTGAAAGTATCAGAATTGATACTATTGATGATTTACATCAAGGA
ATTCAAGAGAAACGAAAATATGAAGTAAATGAAATGAAAATTGTTGAAGCAAAAGAAAAA
GTTTAA
>PvLil2
ATGGCATTAGGAAAAATTTTTTTGAGAACTGATTCTTTCCTCTGCTGCTTCAGATTGGAATCT
GGCGGTATTTTTATTGGAGGTCTCGGTCTTGCATGGTGTTTGTTCCAACTCATTTCTCAAATA
GTTTTGCTGCTATCTCTTATGGTTGTTGAAGATTTTTGTCCACAAAGACATTATTTTTGGAAC
GATAATCAACCTATTAGACATTTGGGAAATTACTCAAAAGAAATGCAGCATGATGTCAAAA
ATGTTACAAGCATGATTCAACGAGGATTACAAAATGCCACACAAACAGCTCAAGAAAAATT
ATATGAAGTCACAAATGAAGAATTTTCATGCACACAAGTCAGCAAAATTCCTCTTGGTCTGA
TTTTGTTAATTGCAATCATTCTCAATGTCATCGGTCTTATTGCGCATTATAGATTGGTTAAAG
GCGTTGAGGAGTCTGATCATTCAAAACTCCCATTAACACTTGGCTACTACAAATTCTTCATTG
GACTCAAATTCATATTTTTGATTGGATTGATAATCTGGGCTTGCTTCAATTACAAATTGTTCA
TGCCAGCAATTTTGATGTTGATCTGCCTCATCATTGATGTTTACATTTATCGAGTCATTGATA
CACTTCGATATAAATATGAAAATACACTACCATTGAGTGCTCAAGAAAACAGAAGAATCAT
CACAACCGTCAAATATGATGATGACGCTAAAGAAAAATTAACAACAAAGTAA
>PvLil3
ATGGCTTTTGCAAATTATATCAGAGTTGATACTTTTTTATGTTGCTTTAGATTGGAATCT
GGAGGGCTTTTTATAGGAGCTTTGGGTCTTTTTTGTGCAGTTCTTCAATTTATAACACAA
ATTGTTTTGATAATTTCACTCATCATAGTTGAAGATTTTTGTCCTGATGAAAATCTTATT
GATAATTATGGAAGAGTTGTAAGTCAAGCACCAAACACACTTAAAGGTCTTGAAAATCTCAC
AAACAATGAAATAAAATGTGCACAAGTCAGCAAAGTTCCACTTGCAGTTGTATTTTTT
GTTGTGATTTGTTTCAACTTTATTTCTATGATTGCGCATTACAAATTGATTAAAGGACTT
GAAGAGCTCAATTTTGAAAAGTTCCTTCTACCAATTGGTTATTATATTTTCTGCATTGTG
ATGAAATTTTTATTTTTTGCGACAATGGTTATACTTACAATTACAGTATCATTTAAAATG
ATTTTCCCTGCAGTTATTTTATTAATTTTGACACTTATTGATGTTTATTTATTTATTGTT
ATTGATACAATTCGATATAAAATTGAAAATTCAATTCCTCCAAATGTGACAAACACTCCA
ATGACTACAATTCATACAAAATTAGTCGATGCAAATAACAAAGATATTGAAATTCCTGCT
GAAATTACAATTCCTAAAGTATATGTATAA
>PvLil4
ATGGAATTTAAGAATATCTTTAAAGGGAAAAAGTTTTTGTGGTTTTTTGATTTGGACACT
GGTGCAGTTATTATTGGAGCTTTGGGTCTACTTTTTGCTGAGTTTCAACTTGTTCTGGAA
GTTATTTTGTTACTTTATTTCACTTTTGGTAGCAATTTTTGTCTTTGGCAGCATTTTTCT
AATACTTATCTCCATCATGACATTTTCTCAACCAAGACACAAAATGATTTTGAAATTGTT
GCTGATATGATGCCAAAAAATTTGAATGATGTCACAATTAATGATATTTCATGTACCTTA
GTTTATAAATATCCTTTAATCCTAATTTTAATTACTGCAATTTTTTTCAACATTATTTCC
ATGATTGCTCACTATAAAATAGTCAAAGCAGTCGAAGAGCGAAACTCAGCAAAATTTTCATT
ACCTTTAAGCCTCTACAAATTTTTCATAGGAATTAAAACAATTTTTCTTACCATTTGG

ACATTCTTTTCGTTTAAAATAATTTATGTTGGATTTTCAATTTTTATTCTTATTTCTATT
TTTATTGATGTTTATATTTATTCAATTATTGACAGACTTCGAGTCAAATATTTAAATCAA
CCTTCAATAAGCTTCATTATAGCATCACAAATTAATGAAGAAATTGAAAATAAAGTTGAT
ATTGAAGATGAAAAAACTGGATTTTTGTTACATTTTTGA
>PvLil5
ATGGAACTAAAAAGTATATTAAAATTTGATGCATTTCTATGCCTCTTCGATTTGGAGTCATGT
GGAATTATTATTGGAGCTTTGGGTCTGCTGTTTGCCTTTTTTCAAATCAATGCTAATATTATCT
TGCTGCTCTTTCTATTTTTTGCTGGGAATTCTTGTCTTCAGCAATATTTTACTGATGGTAGCTT
TATTCGTAATATATTTGCAAAAGGAGCACAAAATGGTTTTGAAAATTTTACTGGTATAGCAG
AAGAAGAAATTAAAAATATCGTAAATTCTGAAAATTCGTGTTCTAACTTTAGTAAATTTGTT
TTTGCTTTAATTCTAATTGGTGGAATTTTCCTGAACGTAATTTCCATAATTGCTCACTACAGA
ATAATAAAAGGTGTCGAAGAGTACAACACTTCGAGATTTCACCTGCCATCGATGTACTACAA
ATTTTTCATAATAATCGAGGGAATTTCATTATTTTTACTAGCAATTTGTAGTTTCTTTTCTTTT
GCAATGTTTGTAGCTACAATTTTAACTTTGATTTTCTTTGTAACTGACATTTACAATTACATA
ATTATTGAAAAACTTCGAATCAAATATTTAAGTCAGCCTCATATAAGTCTCATTTTTATACCA
CAAAATTTGAAAAAACCTAAAAATCAGAAACAAAAAGAAAAGAAAAATAAAAGAGTTGAG
GAATTAAAAAAAGATGAGAAAAATGAAAATTTTTGTTGTCTTTTTGAACACAAACCACCGAC
AAACAATTATTACAAACCACCAACGGATGACCACTATAAGCCGTCCAATAAGAAAGAAGAT
AAAAATAATGACAATATTGATGAAGTTGTTTGTGTTAATTAA
>PvLil6
ATGGCATTTGCAAATTTCTTTAAATTTGATAATTTCTTGGGTATTTTTAAATTAGAATCTGGT
GGCACTATTATTGGAGCCATCGGGTTATTTTACGCGCTCTTTCAATTTGTCATGCAAACAATT
TCATTCTTTTCAATACTTTTTATTCGTGATTTTTGCCCGCAAAAACATTTCATTGATGACGCTA
TGAGCGTTGGTAATCTTCCAAATGAAATTAAACCCGACATAAAAAATATCACAACATATGCA
CAAGAAGGATTACAAAATATTACACATAAAGTTCAAGAAGGAATAAATGATGTCACAGGAA
ATGAAGTTTCATGCACTTTTGTCAGTAAAATTCCATTCATAATTGTTTTAAGTGTTTTGATTG
CCATAAATCTGGTCAGCATGATCGCTCATTATCGATTAATTAAAGGAGTCGAAGAATTCGAC
CCAAGGAAGTTTGAACTTGCAAGAGGCTTTTACTTATTTTTCATTGTAGTCAGATTTATATTG
ATGGTAATCGCAGCTGGTTGGACATTCTTTTCATTCAAAATGATTTATTTAGCACTTACACTT
TTAATTCTTTTGTTAATTGACTTTTATATTTACTCCATAATTGACAGTCTTCAAGATAAATATG
AAAATTCTCTTCCATTGAATGTTGCAACACAAAATATTCATATGAAAACAGTAATTGGTCAA
CCTGTAGATTCACGGAATCAATATCATGTAAATTATTAA
>PvLil7
ATGAGTATAATAGATGTTATGAAAGTAGATAATTTTCTTGGCTGTTTTAGACTTGAATCT
GGTGGATTTTTCATTGCAACCTTCGGATTACTCACAGGATTTCTTCAACTTGTCGGACAAATA
ATTTTTGCAATTTTTCTTATTTTTGTCGAGGATTTTTGTCTTCAGAATCATTTCCTTATTGATG
ACCGAAATAATTATGAAATACAAGAACACATTTCAAGATTGACAAATTTTACACAAGAAGG
ATTGAAAAATGTCACGGATATTGAATTTTCATGTGAAGAAATCAGCAAAACTCCTTTAATTA
TTGCATTAATTTTTGCAATAATTTTCAATACAATTGCTATATTTGCACACTACAGATTGATTA
AAGGACTCGAAGAATTAAATTTTCAAAAATTCATCTGTGCCATTGGCTTCTACATTTTCTGCA
TTGTTTACAAAATTCTATTTTTCATCATGTCAGTTTTGCTGTCGATTTTTGTGTCCTACAAACT
TTTGGTTCCTTCAGTCATTCTCTTAATTCTTACATTTATTGACACTTATATGTTCATTGTGATC
AACACGATTCGCGTAAAAATTGAAAATCCTCCACATTTGACAGTCACACGCACACCTACGAC
AACATTACAAGCTAAACTAGTTGACCTTTCTAATACAGATATTGAAACTCCAGTCAGACTTA
TTGTACCAGAATAA
>PvLil8
ATGGTATCAATCAATATTTTAAAAACCAAAAATTTCCTTGGAATTTTTAATATTGAAACGGG
AGGAATTTTTATTGGATTAGCTGGTTTATTTTGGGCGATTGCACAACTTGTTGCAGAAATAAT
TTTAATACTTTCTCTCGCTGTGGTTGAAGACTTTTGTTTAGAGCGACAAATTTTCTATGCTGA
AGAAACTCTTGGAAATTATTCTGAAGATTTCAAAAATTTCACCGAATTTGCACAACAAGGTT
TACAAGATTTAACATATCAAGCACAAGAAGCATTGTTTAATGTTACCAATACTAAACTTTCA
TGCACACAAATCAGCAAAATTCCAATCGGAATTTTCCTGGTGTCTGCAATAATTTTAAATGTT
GTCGGAATTTTTGCACATTTAAGATTAATAAAAGGTGTAAATGAGCGAGATTCTTCAAAATT

AACTTTGGCAAAAAACTTTTACAAATTTTTCATCATTTTCAAATCAGTTACTTTGATGTTGCT
GACAATTTTTAATTATTATTCCTATGTCATGATAATCTTTACTGTTTTATTTTTATTATTTTTGT
TCATTGATCTTTATGCATATTTGATTATCGAAATTCTTCGTCAAAAATATGAAAATTCTTCCC
AAAATGGAAATGTTATGAGCACACCAAAAAAGAAATCAAAAACAAAAAAGCACCCCAAGC
AGTTCAAATAA
>PvLil9
ATGGCAATCAGAAACTTTTTTGGTAGTGACAAATTTCTTGGACTCTTTTCTTTGGACTCTGGT
GGTATTATCATTGGAGCCCTTGGATTCTTCTGCGGACTTGTCCAATTTATTTCACAACTTGTT
CTACTCTTCTCACTCCTTTTCGTAAAAGATTTTTGCCCACAACGTCATTTGCTTGATGAAACC
ACAAATATTGGAAATTTGCCTCAAGGTACTCAACAAAACATAAAAGACACATTCAATTATGC
TCAACAAGGCATTCAGAATCTCACACGTACAGTGCAAGATAGCTACAAAAACATCAAAGAT
ACTGATTATTCATGCACTTTTGTTAGCAAAATTCCTCTCGTTTTGATTTTGCTCGGTTTAATTT
TGATCAATCTTTTCGCCATCATTGCGCACTACAGATTGATTCGTGGAATTGAAGAGAATGAT
CACAAGAGATTGCGTTTGGCTCGTATTTTCTATCTTTTCTACATTGGATTCCGTTCAATTTTGA
TTATTGCTTTCATAATTTGGGGCTTTTTCAACACAAAAATGTTTTGGGCTGCAGGAGTTTCTT
TGATTTTCCTTTTCATTGATCTCTACATTTATTCAGTCATTGACCATCTCCGCAATAAATATGA
ACATCCACCATTGAATCCACCATTGCATGCAACTCAAACTACTCAGGTTGTTCGAACAAGAA
TTGCTGGAACTATTGATGAGTAA
>PvLil10
ATGGCATTTCCAAATTTTTTCAAAGCTGATAATTTCCTTGGTATTTATAAGTTGGAATCT
GGTGGAATTATTATTGGAGCCGTTGGAATATTTTGGGCACTTCAACAACTTATAACTGAA
ATAATTTTACTTTTTGCTTTATTTTGCCTTCGTGATTTTTGTCCTCAAAGATATTTTATT
AGTGATGAAACATACCTTGAAAATTTCTCAAATGAAACTCAGCACGAAATAAAAAATTTC
ACAAATTATGCACAACATGGATTACAAAATATTACACATAAAGTTCAAGAAGGAATAAAT
GATGTCACAGGAAATGAAGTTTCATGCACTTTTGTCAGTAAAATTCCTATCATTGCTGTT
TTGGTTTTGGCTATTTTAGTGAATATTCTCAGTATTATTGCACATAATAGATTGATTAAA
GGAATTGAAGAGTTGAACCCAGCAAGATTTCAATTAGCAAGAGTCTTTTATCAGTTGTTT
ATTGTAATCAGAGTTATATTTTTGGCAATTGTTGCAATTTGGACATTCTTTTGGCTTAAA
ATGTTTTTAGCAACAATTTTATTTTTATTCCTTTTTTATATTGATTTCTATATTTATTCA
GTTATTGACAAACTTAGTGTTAAATATGAAAAGTCAATTCCTTTAAGTGTTGAGGCTCAA
AATATTCAACTTAAGAGAAAAGTTCCACGTTCTGATTTACCAGAAAGAGTTCAAATTTAA
>PvLil11
ATGGGATTAAAAAAGTTCATTAAAGACGATAGTTTTCTTGGGTTCTTTGATTTGCAAACT
GGTGGAATTATTATTGGAGCCATTGGACTGTTTAGTGCTATTGTTCAAATTACTACAGAA
TATATCTTGCTACTTTCTCTCTTTTTTGTGGATAGTTTGTGCCTTCAGCTTCATTTTCCT
AAGAGTAATTTCAATCTTAATATTCTTGCAAAGAGTACAGAAGATGATATTGCGAATGTC
ACTGATTTAATTCAAACTTTTACAAACAAAGATGTAACATGTGGAGATACCGATAGAATT
CCTTTGGCTTTAATTTTGATTGCTGGAATTTTAATCAACATAACATCAATCATTGCTCAC
TACAGAATCATTAAAGGTGTCGAAGAGCATAATGCAACAAGATTTTCATTTGGTCTCAAA
TATTATAAATTTTTCATTGGTCTTCGAGGATTTTTATTGATTCTTCTGATAATTTGGAGC
TTTTCATCAATTAGATTTATTTTTTATGCATTTTCAATGTTGGCACTTCTTGTAACTGAC
ATTTATATTTATATAATTCTTGATAAACTTTGGGAAAAATATTTAATTCATCCTCCCAAA
AGTATTATAGTTCTTCCTCCTGTAAGTCAAGTTCCAAATCATCATGAAGATCTTTTTAAT
CTTAATAATTTTGGAAAAACTGAAAATGAAATGAATGGTGCTAAAAGTAATTTAGATGAA
AGCAAAAGTGATAGTAGAGAAAGTTTTCGATATATTTTTGAAAATGAATCACCGACAATG
ACAAAAATTGAACCACAAAAAAACATAGAAAAACCAAGAATTCAAGAAAATATTTCGGAA
GTTTTTATTATTGATAACAGTAAGAAAGATAAAATCTCAATTTCAACTTAA
>PvLil12
ATGACAATAGTACAATATTTACGAGTTCAAAATTTTCTTGGACTTTTCAATCTTGAATCT
GGTGGAATTTTAATTGGAGCTTTGGGTGTTTTGATTGCTTTTCTGCAATTGATAGCACAA
ACAGTATTTATTGCTTCACTCTTATTTATTGAAGACTTTTGTCCACAACAGCAATACATT
GATTTCAATTTCAAAGATTCTCCACCAGAAAATTTACAAGATGCCACAAATTGGGTAAAA
GAAAGTTTTCAAAATGTTACAAATGCGACGCAAGAAAATTTACAAGACCTAACATATCTA

GGACAAGTTAATTTTAAAAATGAAACTGGTACAAAATTAGAATGCTCACAACTTGATAAA
CTGCCATTGATTTTGGTGTTTTTCTTCGCAATTTGTTTCAATGCTGTTGCAGTAATTGCG
CACTATAGAGTGATCAAAGGAATTGAAGAGATGAATTTCCGAAGATTTCTTCTTGCAATT
GGTTATTACGTTTTCATGCTAACAGTGAAAGCACTTACATTTGCAGTATTATTAATTCTG
ACGGTTTTTGTATCAATTTACATGCTCATTCCAACAATATTCTTTTTGATTATGATAATA
ATTGATGCATACATTTTAATTGTCATTGATACCATTCGATATAAACTTGAAAAATCTCCC
TATTTGGTTGTAGCACGATCACCAACCACAACAATTGAAACAAGACTATTTGAAGTTCCA
AATGATGATGGTGAAGCTGCAAGAATTATTATACCAAAATTATTGGATTAA
>PvLil13
ATGAAGGTCAGAAATTTATTTGGAGGCGATAGCTTTCTTGGACTTTTCACATTGGATACTGG
CGGAATAATTATTGGAGCACTTGGATTATTCTGCTCATTAATTTCGCTCGTTGCAAACTCATT
CTTCTTATTTTCACTCTTATTTGTGAATGATTTTTGTCCTCAACGTTACTTCATTGAAGACAAA
GATGTTGGATATTTCTCAAAGGAAACTCAACAAACTATCAAAAATACTGTGAATTATGCACA
AGAAAGTATCCAGAATCTCACACGACAAGCACAAGGTGCTTTGACAAATGCTACAGACAAA
GATATTTCATGCACTTTTGTCAGCAAAATTCCATTCTTCTTGATTTTCCTTGGCATGATCATCC
TCAGCATTATTGGTGCCATCGCTCATTACAGATTGATGAAAGGAATTGAAGAGAATGACCAC
AAGAAAACACGTTTGGCTCGTGGCTACTACATGTTTTACATTGGACTCAGAGCAATTTTATTC
ATTGTCTTTTTGATTTGGTGTTTCTTCAATGGCAAAATGCTTTGGCCTGCAATTTTCTCTTTGG
TCCTTCTTCTTATTGACCTCTATGCTTATTCTATAATCGATAAACTTCGCGTCAAATATGAGC
ACACACCTCCAGTTAATACTGCACAAACAACGCAATTCGTTCAACGAACAAGAATCACTGCT
TGA

Supplementary Data 1

Nucleotide sequence of non-coding splice form of *PvLil-4* gene in FASTA format

>PvLil4_T2
ATGGAATTTAAGAATATCTTTAAAGGGAAAAAGTTTTTGTGGTTTTTTGATTTGGACACT
GGTGCAGTTATTATTGGAGCGTGAGTTAGAAATTCGATAAAAAGTTTCTAAAATTTTCGA
AGTTTGGGTCTACTTTTTGCTGAGTTTCAACTTGTTCTGGAAGTTATTTTGTTACTTTAT
TTCACTTTTGGTAGCAATTTTTGTCTTTGGCAGCATTTTTCTAATACTTATCTCCATCAT
GACATTTTCTCAACCAAGACACAAAATGATTTTGAAATTGTTGCTGATATGATGCCAAAA
AATTTGAATGATGTCACAATTAATGATATTTCATGTACCTTAGTTTATAAATATCCTTTA
ATCCTAATTTTAATTACTGCAATTTTTTTCAACATTATTTCCATGATTGCTCACTATAAA
ATAGTCAAAGCAGTCGAAGAGCGAAACTCAGCAAAATTTTCATTACCTTTAAGCCTCTAC
AAATTTTTCATAGGAATTAAAACAATTTTTCTTACCATTTGGACATTCTTTTCGTTTAAA
ATAATTTATGTTGGATTTTCAATTTTTATTCTTATTTCTATTTTTATTGATGTTTATATT
TATTCAATTATTGACAGACTTCGAGTCAAATATTTAAATCAACCTTCAATAAGCTTCATT
ATAGCATCACAAATTAATGAAGAAATTGAAAATAAAGTTGATATTGAAGATGAAAAAACT
GGATTTTTGTTACATTTTTTC
